# Locked nucleic acid building blocks as versatile tools for advanced G-quadruplex design

**DOI:** 10.1093/nar/gkaa720

**Published:** 2020-09-05

**Authors:** Linn Haase, Klaus Weisz

**Affiliations:** Institut für Biochemie, Universität Greifswald, Felix-Hausdorff-Str. 4, D-17489 Greifswald, Germany; Institut für Biochemie, Universität Greifswald, Felix-Hausdorff-Str. 4, D-17489 Greifswald, Germany

## Abstract

A hybrid-type G-quadruplex is modified with LNA (locked nucleic acid) and 2′-F-riboguanosine in various combinations at the two *syn* positions of its third antiparallel G-tract. LNA substitution in the central tetrad causes a complete rearrangement to either a V-loop or antiparallel structure, depending on further modifications at the 5′-neighboring site. In the two distinct structural contexts, LNA-induced stabilization is most effective compared to modifications with other G surrogates, highlighting a potential use of LNA residues for designing not only parallel but various more complex G4 structures. For instance, the conventional V-loop is a structural element strongly favored by an LNA modification at the V-loop 3′-end in contrast with an alternative V-loop, clearly distinguishable by altered conformational properties and base-backbone interactions as shown in a detailed analysis of V-loop structures.

## INTRODUCTION

**Figure 1.**
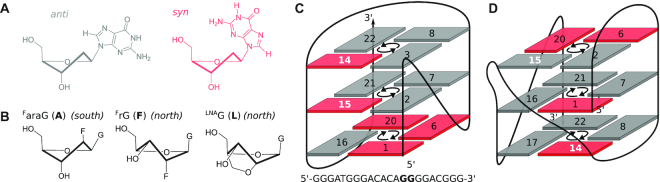
Nucleoside conformation and topology adopted by native and 14,15-modified ODN. (**A**) Glycosidic bond angle. (**B**) *Anti*-favoring G analogs; ^F^araG (left) and ^F^rG (center) prefer sugar puckers in the *south*/*south-east* and *north* domains, respectively, ^LNA^G (right) is restricted to a C3′-*endo* conformation. (**C**) Hybrid-type topology of native ODN. D) ODN-derived conventional V-loop topology. *Anti* and *syn* Gs are represented by grey and red rectangles, respectively. Modification sites are highlighted by white numbers. Tetrad polarity is indicated by arrows.

Quadruplexes (G4) are among the most studied alternative nucleic acid structures both for their potential regulatory roles *in vivo* (1,2) and their promising characteristics for biotechnological applications (3,4). G4 structures can be formed by G-rich sequences through assembly of four G-tracts, enabling the stacking of usually two to four G-quartets. These tetrads, square planar arrangements of Hoogsteen hydrogen-bonded guanine bases constitute the main characteristics of G-quadruplexes. However, with possible variations in G4 molecularity, types of G-tract connecting loops, relative G-tract orientations as well as tetrad polarities, i.e. the directionality of hydrogen bonds within a G-quartet, DNA G-quadruplexes display a remarkable variety of folding topologies (5,6). In spite of the ever growing number of high-resolution structures, prediction or design of a particular G4 fold is challenging, as a variety of factors such as buffer pH (7,8) or the nature of coordinating cations (9,10) may critically influence G4 folding in addition to a heavy dependence on primary sequence (11,12). In this respect, the glycosidic torsion angle determining the relative orientation of base and sugar moieties is of particular importance, as a switch in G-tract orientation or tetrad polarity is always accompanied by a *syn–anti* or *anti–syn* transition (Figure 1A).

A common strategy in targeted G4 design is therefore the introduction of G analogs with particular glycosidic torsion angle preferences for the directed manipulation of G4 topologies (13). Thus, *syn*-favoring G surrogates with a bulky C8-substituent have been used to induce *anti–syn* transitions in parallel qudruplexes, often accompanied by a reversal of the 5′-tetrad polarity (14–17). Similarly, sugar-modified G analogs such as 2′-F-riboguanosine (^F^rG) and its epimer 2′-F-arabinoguanosine (^F^araG) are known to favor the *anti* conformation (Figure 1B). By affecting glycosidic torsion angles, these 2′-modified G analogs often cause a tetrad polarity reversal when introduced in the 5′-terminal G-quartet of hybrid-type quadruplexes (18–21). In particular, substitution effects have been studied extensively for the most malleable ODN sequence *d*(G_3_ATG_3_ACACAG_4_ACG_3_) (Figure 1C) (22), where even the central tetrad polarity could be inverted by introduction of a single ^F^araG or ^F^rG (23). The structural impact of *anti*-favoring G analogs is further complicated as the modification in the sugar moiety primarily impacts sugar conformational features. While ^F^araG residues usually favor a *south*/*south-east* sugar pucker, the *anti*-preference of ^F^rG is thought to be linked to a strongly favored C3′-*endo* or *north*-type conformation in analogy to riboguanosine (rG) (Figure 1B). However, ^F^rG and rG residues in modified ODN structures may adopt *north* or *south* puckers depending on the modification site (19,21,23,24).

In contrast, a V-loop structure F(14,15) generated upon substitutions of ^F^rG but also rG at the two *syn* positions 14 and 15 in ODN’s third antiparallel G-tract was found to exclusively rely on favored *north*-type sugars for modified residues (Figure 1D) (25). The preference for an N-type sugar pucker seems to be the major driving force for this rearrangement, as the incorporated ^F^rG/rG residue in position 14 is forced to adopt the unfavored *syn* glycosidic angle. The interplay of glycosidic torsion angle and sugar pucker grows more and more complex as demonstrated by most recent findings on mixed ^F^rG/^F^araG modifications again at position 14 and 15 of ODN (26). Depending on the order of ^F^araG/^F^rG substitutions, either the conventional V-loop structure with N-puckered modified residues (A14F15) or an alternative V-loop topology with reversed top tetrad based on sugar puckers in the *south* domain (F14A15) was induced (Supplementary Table S1). In both structures, modified residue 14 at the V-loop 5′-end adopts a highly disfavored conformation, unfavorable with respect to both glycosidic bond angle and sugar pucker. Thus, with a variety of combinations of supposedly favored or unfavored sugar puckers and glycosidic bond angles for rG, ^F^rG and ^F^araG residues (Supplementary Table S1), it seems that the preference for the *anti* conformation or a particular sugar pucker is neither absolute nor strictly linked, but strongly depends on the local structural context.

Another *anti*-favoring G analog, the bicyclic 2′-*O*-4′-C-methylene riboguanosine mimic (locked nucleic acid, ^LNA^G) displays less conformational flexibility (Figure 1B). It is strictly locked in a C3′-*endo* conformation by the 2′-*O*-4′-C-methylene bridge, and the very high pucker amplitude of about 60° strongly disfavors a *syn* conformation due to steric clashes between the base and the H3′ proton in the axial position (27). Thus, incorporation of this RNA mimic has been observed to induce structural rearrangements from antiparallel to parallel G4s (27,28), and often caused thermal stabilization of parallel DNA G-quadruplexes (29–33). However, effects of LNA modifications are not always consistently rationalized by a (non-)matching glycosidic angle at the substitution site. For instance, the conformationally constrained ^LNA^G seemed incompatible with *anti*-positions preceding short propeller loops, and substitution for an *anti*-G in the central tetrad of a hybrid-type quadruplex caused disruption of the quadruplex fold (33).

Nevertheless, LNA-modified G-quadruplexes offer great potential for applications in therapeutics and diagnostics not only for their exceptional biostability (34). In fact, an up to 8-fold increase of anti-HIV activity could be observed upon LNA modifications of two G4 forming sequences (35), and a thrombin binding aptamer variant with improved anticoagulant activity was generated by substitution of ^LNA^G for the *anti*-G at the 3′-end of the antiparallel G-quadruplex (36). LNA incorporation was also used to optimize a G4 aptamer in terms of thermal stability and nuclease resistance and the LNA-functionalized G4 was used as an aptasensor in a proposed bioassay (37).

An unusual interlocked G4 structure was formed upon ^LNA^G incorporation into all *anti* positions of a bimolecular antiparallel G4 (38). The resulting four-layered topology was termed V4 fold for its four V-shaped loops, each of them harboring an LNA residue at the V-loop 3′-end. A driving force for this rearrangement may derive from the stabilization of the V-loops by the enforced N-type sugar pucker in the 3′-flanking position as also suggested for F(14,15) (25). Strikingly, the very constrained LNA sugar pucker seems to match exactly the backbone conformational requirements imposed by the 0-nt V-shaped loop. Even though unmodified, Gs at the V-loop 5′-ends also adopt a C3′-*endo* conformation. While induced N-type sugar puckers in the vicinity of incorporated LNA residues are not unusual (27,30,39), the *north* pucker in this case may be due to the intrinsic conformational features of the V-loop. In fact, a prevalence of *north* conformers for flanking residues at both V-loop ends was also found for the majority of monomolecular G-quadruplexes featuring such loops (25).

In an attempt to further unravel conformational features of V-loops and to challenge LNA compatibility with non-parallel G4 structures, we here extend our studies of 14,15-modified ODN to single and dual LNA substitutions as well as combined modifications with ^LNA^G and ^F^rG. In analogy to the V4 structure, an LNA modification in position 15 at the V-loop 3′-end is likely to support the V-loop structure. On the other hand, an ^LNA^G residue in position 14 is expected to be incompatible with V-loop formation as an associated *syn*-^LNA^G is thought to be highly disfavored. While we uncover unanticipated conformational flexibility for LNA residues and an ability to stabilize different non-parallel G4 structures, the locked sugar moiety is shown to be even more effective than its 2′-fluorinated analog(s) in generating particular stable quadruplex folds, underlining the strong potential of LNAs in G4 design for nanotechnological or therapeutic applications.

## MATERIALS AND METHODS

### Materials and sample preparation

^LNA^G and ^F^rG modified oligonucleotides were purchased from IBA (Göttingen, Germany) or Microsynth (Balgach, Switzerland) and quantified after ethanol precipitation based on their extinction coefficient at 260 nm as provided by the supplier. NMR samples were prepared by dialyzing the corresponding oligonucleotide against 10 mM potassium phosphate buffer at pH 7, followed by heating to 90°C for 5 min and subsequent cooling to room temperature. Concentrations of NMR samples were about 0.4 mM. For optical measurements, oligonucleotide concentrations of 5 μM were used either under NMR conditions or in a buffer containing 20 mM potassium phosphate, 100 mM KCl, pH 7.

### Gel electrophoresis and optical measurements

For native gel electrophoresis, annealed oligonucleotide samples (15 pmol per lane) were loaded onto a 15% polyacrylamide gel (acrylamide:bis-acrylamide 19:1). Separation was performed at room temperature in TBE buffer supplemented with 10 mmM KCl using a voltage of 80 V. Bands were visualized by staining with a 50 μM thiazole orange solution.

Circular dichroism (CD) spectra were acquired with five accumulations, a scanning speed of 50 nm/min and a bandwidth of 1 nm at 25°C on a Jasco J-810 spectropolarimeter (Jasco, Tokyo, Japan). All spectra were blank-corrected by subtraction of the buffer spectrum. Melting curves were recorded in triplicate on a Jasco V-650 spectrophotometer equipped with a Peltier temperature control unit (Jasco, Tokyo, Japan) with quartz cuvettes of 10 mm path length. The absorbance at 295 nm was measured between 10 and 90°C in 0.5°C intervals with a heating rate of 0.2°C/min. The melting point *T*_m_ was determined from the minimum of the first derivative of the heating phase.

### NMR spectroscopy

All NMR spectra were acquired on a Bruker Avance Neo 600 MHz spectrometer equipped with an inverse ^1^H/^13^C/^15^N/^19^F quadruple resonance cryoprobehead and z-field gradients. Topspin 4.0.4 and CcpNmr Analysis 2.4 were used for spectral processing and analysis (40). An optimized WATERGATE with w5 element was employed for solvent suppression in 1D spectra and 2D NOE experiments (41). NOESY spectra were recorded with mixing times of 80–300 ms in either 90% H_2_O/10% D_2_O or 100% D_2_O. DQF-COSY spectra were acquired in D_2_O with solvent suppression through presaturation. Phase-sensitive ^1^H–^13^C HSQC experiments optimized for a ^1^*J*_CH_ of 170 Hz were acquired with a 3–9–19 solvent suppression scheme in 90% H_2_O/10% D_2_O usually employing a spectral width of 7.5 kHz in the indirect ^13^C dimension and 512 *t*_1_ increments.

### NMR structure calculations

A simulated annealing protocol in Xplor-NIH 2.49 was used to generate 100 starting structures of the DNA sequence (42). For subsequent calculations with Amber16 (43), partial atomic charges for the modified ^F^rG residue were calculated using the RED software (44) while partial charges and an optimized Amber force field for LNA residues were adopted from Condon *et al.* (45). A restrained simulated annealing was performed in implicit water using the parmbsc0 force field including the χ_OL4_, εξ_OL1_ and β_OL1_ corrections (46–49). Ten lowest-energy structures were selected, equilibrated for 4 ns with explicit solvent, and shortly minimized in vacuum. Structural parameters were determined with the 3DNA software package (50). Details on the calculation process can be found in the Supplementary Material.

## RESULTS

### Initial characterization of 14,15-modified ODN sequences

**Figure 2.**
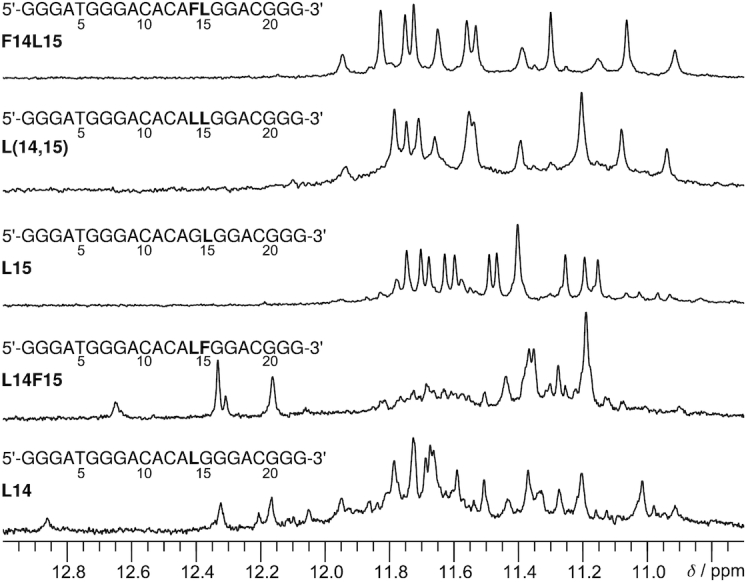
Imino proton spectral region of different 14,15-modified ODN sequences in 10 mM KP_i_ buffer, pH 7, at 35°C. ^F^rG and ^LNA^G modifications are represented by letters F and L, respectively. The elevated temperature was selected for comparison as some imino resonances are strongly broadened at lower temperatures especially for F14L15 and L(14,15).

^LNA^G and ^F^rG residues were incorporated into positions 14 and 15 of the ODN sequence to obtain two single LNA modifications (L14 and L15 with ^LNA^G in position 14 and 15, respectively), two mixed modifications (F14L15 and L14F15, with ^F^rG and ^LNA^G at position 14 and 15 and vice versa) as well as the LNA disubstituted L(14,15) (Supplementary Table S1). The five different 14,15-modified ODN sequences were initially characterized by gel electrophoresis and 1D ^1^H NMR spectroscopy (Figure 2 and Supplementary Figure S1). Observation of a single band migrating close to native ODN suggests a monomoleculer structure for all modified sequences. Also, G-quadruplex formation is indicated by the presence of guanine imino resonances in the 10.7 to 12 ppm region, characteristic of Hoogsteen hydrogen bonding. Disregarding low-intensity signals arising from minor species, twelve major G4-characteristic imino resonances point to the formation of one three-layered quadruplex species for all modified sequences with ^LNA^G in position 15. In contrast, a larger number of overlapping signals suggests considerable polymorphism for L14 and L14F15. Of note, several downfield shifted signals in the 12–13 ppm region point to the formation of Watson-Crick GC basepairs in addition to G-tetrads. Unfortunately, the resolution and number of imino signals hardly improved upon varying the temperature (Supplementary Figure S2). Consequently, a determination of any of the adopted topologies through 2D NMR methods proved impossible, thus L14F15 and L14 modifications were not further investigated in detail. F14L15, L(14,15) and L15, on the other hand, were additionally characterized by circular dichroism (CD) spectra, all showing a negative band around 240 nm along with two positive signals around 263 and 290 nm, characteristic of a G4 fold featuring both homopolar and heteropolar stacking interactions between G-quartets (Supplementary Figure S3). Thermal stabilities were also determined by UV melting experiments and will be discussed below along with the structural details (Supplementary Table S2).

### F14L15 adopts a V-loop topology

**Figure 3.**
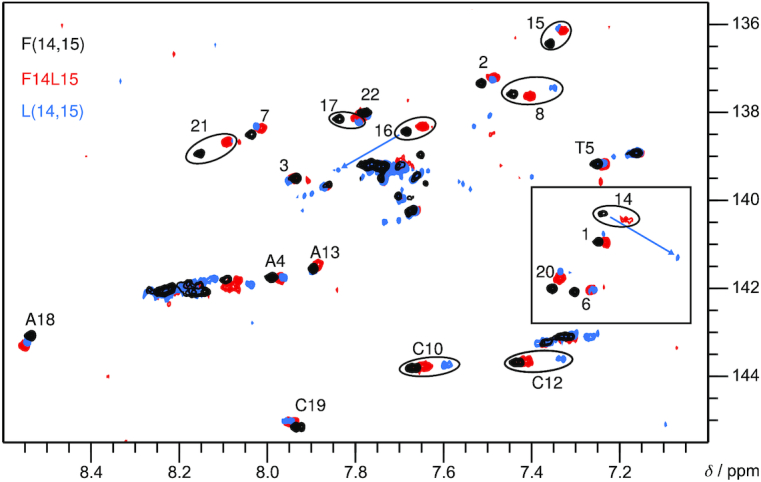
Superposition of H6/H8-C6/C8 regions from ^1^H–^13^C HSQC spectra of F14L15 (red, 0.4 mM), L(14,15) (blue, 0.4 mM) and F(14,15) (black, 1 mM) acquired at 35°C in 10 mM KP_i_, pH 7. *Syn* guanosines including ^F^rG/^LNA^G14 are framed by a rectangle. Unlabeled signals arise from unfolded or minor species. Most pronounced shifts for residues 14 and 16 of L(14,15) are indicated by arrows.

Characterization of the F14L15 topology was easily accomplished due to a pronounced similarity of 2D NMR spectra to those of F(14,15) adopting the V-loop structure (Figure 1D). Thus, the almost perfect superposition of crosspeaks in ^1^H-^13^C HSQC spectra of F14L15 and F(14,15) strongly suggests formation of the same topology for these two modified sequences (Figure 3). The four *syn* Gs of the V-loop structure are not only identified by their characteristically downfield shifted ^13^C8 resonances, but also by unusually strong intraresidual H8–H1′ NOE crosspeaks (Supplementary Figure S4). Supported by a very good dispersion of resonances, sequential connectivities can be unambiguously traced within all G-tracts in the H8–H1′ and H8–H2′/H2” region. Residues of the first lateral loop are linked to the preceding G-tract through sequential contacts enabling their straightforward assignment. In the absence of further clear contacts to loop regions, all other loop residues with the exception of unassigned A9 and A11 were identified based on a comparison to F(14,15) (Figure 3). Being in full agreement with the V-loop topology, all G-core imino protons were unambiguously assigned through H8-H1 as well as H1-H1 NOE contacts and additionally confirmed by H–D exchange experiments (Supplementary Figures S5 and S6, Supplementary Table S4).

The 2D NOE spectra feature several indicators for very close structural similarities between F14L15 and F(14,15). These include (i) similar contacts of outer tetrad imino protons to loop residues (Supplementary Figure S5), (ii) a reverse H8_i_-H1′_i+1_ sequential contact between ^LNA^G15 and G16 in the absence of a typical sequential H8_i+1_-H2′_i_ contact (Supplementary Figure S7), (iii) a non-conventional H8_i_-H2′_i-2_ contact between G16 and ^F^rG14 (Supplementary Figure S7), (iv) an unusually downfield shifted H3′ proton for ^F^rG14 (Supplementary Figure S8), and (v) ^F^rG14 vicinal F2′-H1′/H3′ scalar couplings in perfect agreement with values obtained for F(14,15) (Supplementary Figure S8) (25). Thus, with a (*north*,*syn*) combination for ^F^rG14 and an *anti*-^LNA^G locked in the C3′-*endo* pucker, conformations of V-loop flanking modified residues in F14L15 completely match those in F(14,15).

### LNA *syn* conformer is part of the L(14,15) V-loop structure

For L(14,15), assignment is hampered by a number of overlapping H8 and H1′ resonances, yet sequential NOE walks can be traced within all G-tracts (Supplementary Figure S9). Interestingly, the H6/H8-C6/C8 region of a ^1^H–^13^C HSQC spectrum again matches well with F(14,15) with larger chemical shift perturbations only observed for modified residue 14 and G16 following the two modification sites (Figure 3). Of note, four residues display C8/H8 resonances in the region typical for *syn* Gs. Surprisingly, the most upfield shifted H8 resonance at 7.06 ppm is assigned to ^LNA^G14 adopting a *syn* glycosidic angle. While in full agreement with the ^F^rG14 conformation in F(14,15) and F14L15 and a strong indication for a V-loop topology also for L(14,15), this *syn* conformation for an LNA nucleotide is highly unexpected and to the best of our knowledge unprecedented. Additional support for the unusual LNA *syn* conformer comes from a strong intraresidual H8–H1′ NOE crosspeak and a missing H8-H2′ contact, reflecting corresponding short and long intranucleotide H8–H1′ and H8–H2′ distances in a *syn* conformer (Supplementary Figure S10).

Other characteristic features of the *syn* residue 14 in the V-loop structure relate to an unusually downfield shifted H3′ proton and a non-conventional H8_i_-H2′_i-2_ contact between G16 and residue 14 (see above). While ^LNA^G14 H3′ could not be unambiguously assigned, it is likely isochronous with the H2′ resonance at 4.89 ppm in line with H3′ chemical shifts of ^F^rG14 in F(14,15) and F14L15. The observed unusual NOE contact between G16 H8 and ^LNA^G14 H2′ and another V-loop characteristic crosspeak between ^LNA^G15 H8 and G16 H1′ are subject to some ambiguity due to overlap with other NOE crosspeaks (Supplementary Figure S10). However, the absence of a typical sequential H8-H2′ contact between ^LNA^G15 and G16 reflects the strand polarity inversion within the third G-tract, also identified as a characteristic structural feature of the conventional V-loop topology (25).

Unfortunately, only few imino resonances give rise to correlations in 2D NOE spectra with sometimes severely broadened signals of low intensity, attributable to a rather flexible G-core with a non-matching ^LNA^G14 conformer as well as to partial unfolding as a result of only moderate G4 thermal stability (Supplementary Table S2). Nevertheless, combined assessment of H-D exchange experiments, NOE crosspeaks involving imino resonances, and comparison with assigned F14L15 chemical shifts enabled imino proton assignments for L(14,15) (Supplementary Figures S6 and S11, Supplementary Table S5). Thus, although limited in number, H1–H8 and H1–H1 NOE contacts are in full agreement with expected hydrogen bonding patterns and relative tetrad polarities, further confirming that L(14,15) adopts a stable V-loop structure in spite of a most unfavorable *syn*-^LNA^G14.

### L15: Complete rearrangement to an antiparallel topology

**Figure 4.**
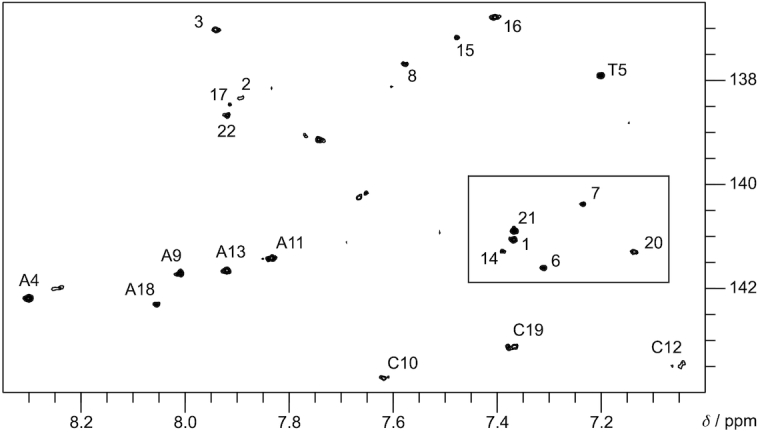
H6/H8-C6/C8 region from a ^1^H–^13^C HSQC spectrum of L15 (0.4 mM) acquired at 25°C in 10 mM KP_i_, pH 7. *Syn* guanosines are framed by a rectangle.

**Figure 5.**
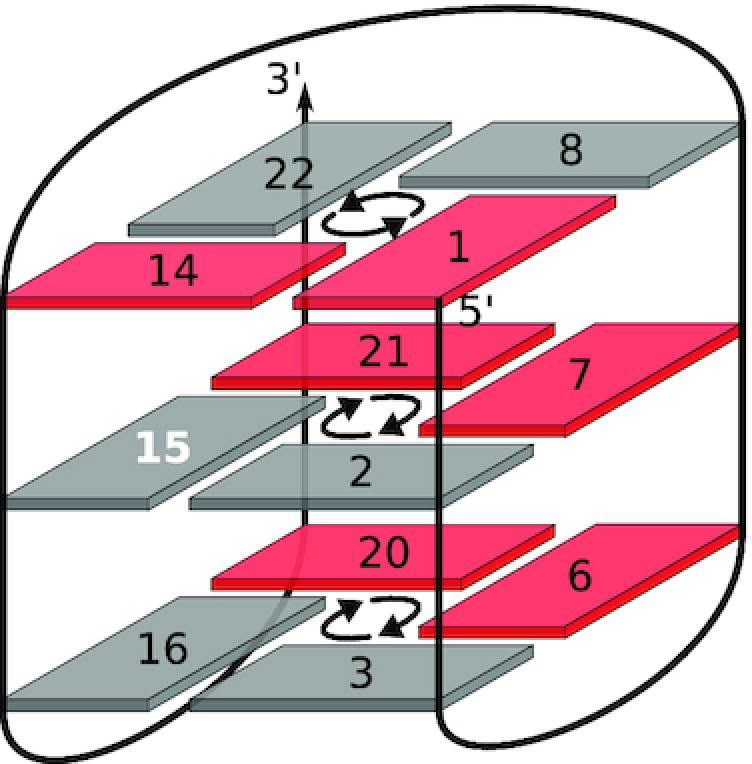
Schematic representation of the L15 topology with its reversed central tetrad polarity and inverted first G-tract. *Anti* and *syn* Gs are represented by grey and red rectangles, respectively.

The adopted topology for L15 differs from the V-loop structure as is clearly apparent from the ^1^H–^13^C HSQC spectrum with six Gs exhibiting C8/H8 resonances in the typical *syn* region (Figure 4). G-tract assignments were easily accomplished in 2D NOE spectra due to sequential contacts extending into loop regions and the characteristically downfield shifted H2′-resonance of ^LNA^G15 as a convenient starting point (Supplementary Figures S12 and S13). Here, the six *syn* conformers are confirmed by strong H8-H1′ NOE contacts, and two *syn-syn-anti* along with two *syn–anti–anti* G-tracts were identified based on characteristic H8_i_–H1′_i+1_ NOEs for *syn-syn* steps and a rectangular pattern of H8_i_–H1′_i-1_ and H8_i_–H1′_i+1_ NOEs for *syn–anti* steps (Supplementary Figure S13). Thus, L15 comprises the same four G-tracts as the native hybrid-type quadruplex (Figure 1C) but with changed glycosidic bond angles for the central tetrad in all but the first G-tract. Apparently, the LNA modification in position 15 without additional modification in position 14 triggers the same rearrangement as observed for corresponding modifications with ^F^rG and ^F^araG (23). Thus, a combined reversal of central tetrad polarity and first G-tract orientation results in the antiparallel topology depicted in Figure 5. Relative tetrad polarities and hydrogen bonding patterns for this topology are confirmed by the expected sets of H1–H1 contacts and almost complete H8-H1 contacts, respectively. These enabled a straightforward assignment of all G-core imino resonances in agreement with H–D exchange experiments (Supplementary Figures S6 and S14, Supplementary Table S6).

### High-resolution structures of F14L15 and L15

**Figure 6.**
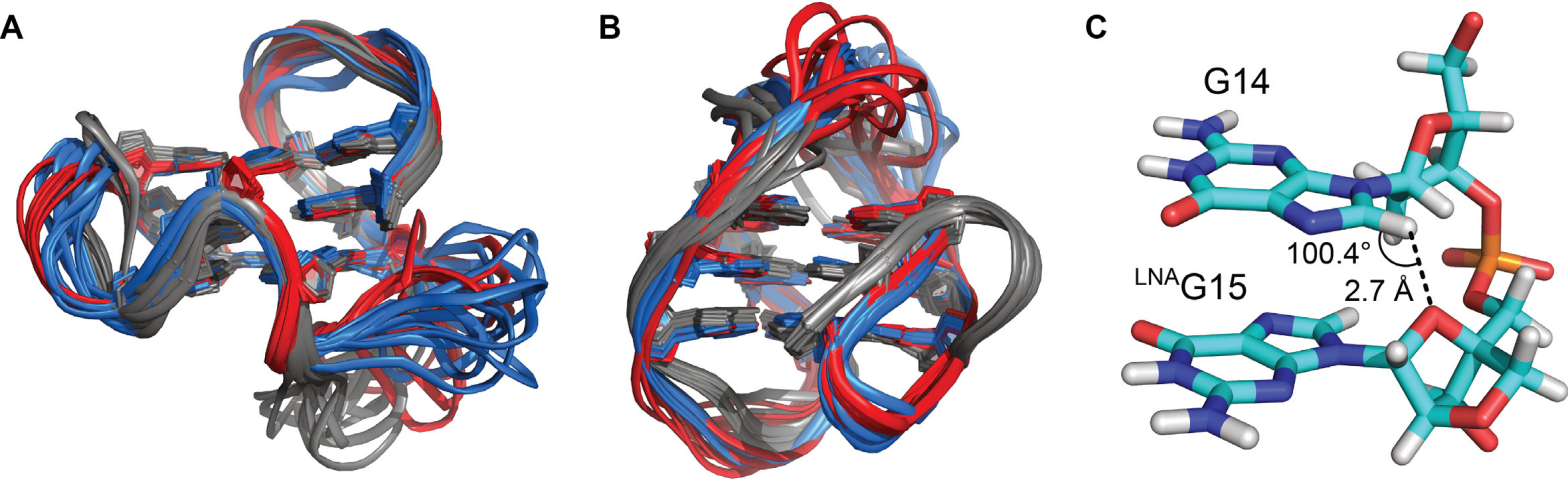
High-resolution structures. (**A**) Superposition of the structural ensembles of F14L15 (red), A14F15 (blue, PDB ID 6TC8) and F(14,15) (gray, PDB ID 6RS3). (**B**) Superposition of the structural ensembles of L15 (red), antiparallel A15 (blue, PDB ID 6F4Z) and native ODN (gray, PDB ID 2LOD). (**C**) G14–^LNA^G15 fragment in a representative structure of L15 showing the short ^LNA^G15 O4′–G14 H8 contact and the corresponding O4′–H8–C8 angle.

For a deeper structural insight, high-resolution structures of F14L15 and L15 were determined by molecular dynamics calculations in explicit water using NMR distance and torsion angle restraints (Supplementary Table S3, Supplementary Figures S15 and S16). A superposition of F14L15 with the two analogous ODN derived V-loop topologies of F(14,15) and A14F15 shows an almost perfect agreement in global structure with only minor deviations in conformation and degree of flexibility for lateral and propeller loops (Figure 6A). The local V-loop conformation agrees perfectly between F14L15 and F(14,15) as already suggested by the observation of characteristic spectral features. Thus, short ^LNA^G15 H8–G16 H1′ and G16 H8–^F^rG14 H2′ contacts are in agreement with observed NOE crosspeaks and only slightly larger than corresponding interatomic distances in F(14,15) (Supplementary Figure S17A). Of note, the N-type sugar pucker of V-loop flanking modified residues is highly conserved between F14L15, F(14,15), and A14F15 (Supplementary Figure S18A). Among the three structures, a difference in type of G analog apparently only causes small deviations in the pseudorotation phase angles (PPA). Thus, a reduced PPA for ^F^araG14 in A14F15 may be required for formation of the stabilizing F2′· · ·H–N hydrogen bond (26). On the other hand, ^LNA^G15 in F14L15 cannot mimic the C4′-*exo* conformation adopted by corresponding ^F^rG residues in F(14,15) and A14F15 for geometric reasons. However, as previously seen in the V4 structure (38), the fixed LNA sugar pucker seems to perfectly match a most favorable backbone conformation at the V-loop 3′-end as also indicated by a very low G-core pairwise heavy atom RMSD of 0.54 Å and high thermal stability of F14L15 (Supplementary Tables S3 and S2).

For L15, the major difference to native ODN is the reversed orientation of the first G-tract, easily visualized by the conversion of the first propeller to a lateral loop (Figure 6B). On the other hand, the topology of L15 is identical to the rearranged A15 structure hosting an ^F^araG residue in position 15 instead of ^LNA^G. Within the G-core, local structural differences are mainly observed in the sugar pucker at the modification site. While ^LNA^G15 is locked in the C3′-*endo* conformation, ^F^araG15 adopts a preferred *south-east* pucker (Supplementary Figure S18B). In contrast to previous observations that LNA incorporation may induce *north*-type puckers especially for 3′-neighboring DNA residues (27,30,39), the impact in this case is locally restricted to the modification site as G14 and G16 adopt a sugar pucker in the *south* domain just as in antiparallel A15 and native ODN. In fact, the sugar conformation of G16 in L15 is very well defined and actually shifted to a higher rather than a lower PPA value.

While the antiparallel A15 and the corresponding structure carrying an ^F^rG analog in position 15 were found to be stabilized by an intraresidual and sequential F2′· · ·H8-C8 hydrogen bond, respectively (23), the O2′ of ^LNA^G15 is turned away from any neighboring H8 as a consequence of the enforced C3′-*endo* conformation. On the other hand, O4′ closely approaches G14 H8 with an interatomic distance of about 2.7 Å and O4′-H8-C8 angles in the range 91° to 104° (Figure 6C and Supplementary Figure S19A). We wondered whether such an unusual base-backbone interaction was LNA-specific and if it could also be observed in a different structural context. Looking at ^LNA^G15 in F14L15, the O4′ is indeed turned toward the H8 of a neighboring G. However, as a consequence of the unique V-loop architecture and the strand polarity inversion between ^LNA^G15 and G16, O4′ does not interact with the 5′- but rather with the 3′-neighboring G16 H8 (Supplementary Figure S17B). Noticeably, the O4′–H8 interaction seems to compete with an alternative O5′-H8 hydrogen bond observed in about half of the states of the structural ensemble (Supplementary Figure S17C).

## DISCUSSION

### LNA-induced stabilization: thermodynamic aspects

**Figure 7.**
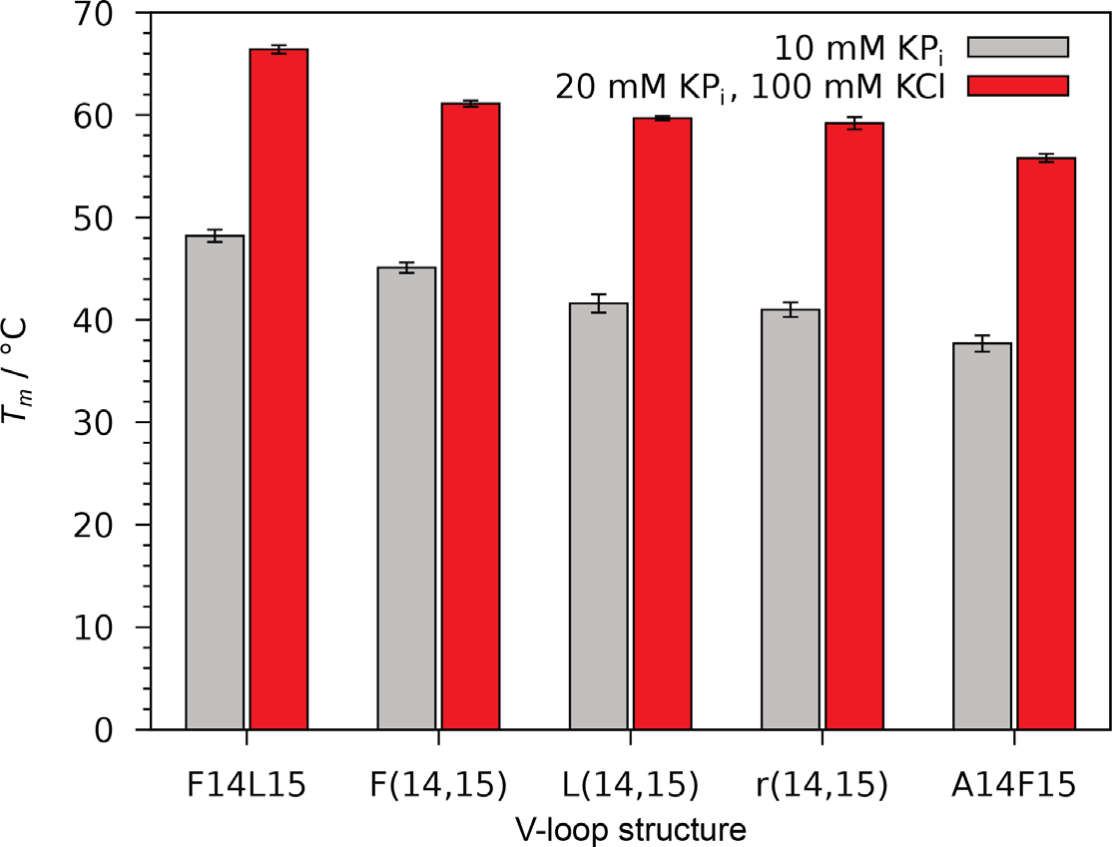
Melting temperatures *T*_m_ for the different ODN derived V-loop structures F14L15, L(14,15), F(14,15) (25), r(14,15) (25) and A14F15 (26) in low- and high-salt potassium buffer, pH 7. Error bars show the standard deviation of three independent measurements.

Two distinct G4 folds can effectively be stabilized upon an LNA-modification in position 15 of the hybrid-type quadruplex ODN. In combination with a *north*-favoring G analog in position 14, refolding to a V-loop topology harboring ^LNA^G15 at its V-loop 3′-end is induced. In addition to F14L15 and L(14,15), three other 14,15-modified ODN sequences have previously been shown to adopt the same V-loop structure: dual-modified F(14,15) and r(14,15) as well as mixed modified A14F15 with a *south*-favoring ^F^araG at the V-loop 5′-end (Supplementary Table S1) (25,26). All structures are stable at ambient temperatures in spite of the disfavored *syn* conformation of the incorporated G analogs at the V-loop 5′-end. Thermal stabilities in both high- and low-salt potassium buffer decrease in the following order: F14L15 > F(14,15) > L(14,15) ∼ r(14,15) > A14F15 (Figure 7).

Among the 14,15-modified V-loop structures, L(14,15) has only a moderate thermal stability due to a most unfavorable *syn* glycosidic torsion angle of ^LNA^G14. Surprisingly, r(14,15) harboring more flexible rG residues is of similar stability and A14F15 with a (*north*,*syn*) conformation for ^F^araG14, disfavored in terms of both sugar pucker and glycosidic angle, is even less stable. Apparently, the contribution of (un)favorable sugar puckers results in a strong energetic penalty for the disfavored N-type pucker of ^F^araG14 in A14F15. Most importantly, the fixed C3′-*endo* pucker of LNA residues in position 15 seems to stabilize the V-loop more than conformationally less restrained ^F^rG or rG as indicated by an increased thermal stability of F14L15 relative to F(14,15). On the other hand, an LNA *syn* conformation is expected to be strongly disfavored due to its fixed C3′-*endo* pucker and the high pucker amplitude. A matching LNA sugar in position 15 apparently compensates for the highly unfavorable *syn-*^LNA^G conformer in position 14, explaining comparable stabilities of L(14,15) and r(14,15). Thus, a strongly favored ^LNA^G residue at the V-loop 3′-end even allows for a strongly disfavored LNA *syn* conformer at the V-loop 5′-end.

While ^LNA^G15 plays a V-loop stabilizing role in combined 14,15-modifications, its effect without any additional modification in position 14 is quite distinct. The single LNA modification in L15 triggers a complete refolding from the native hybrid-type structure to an antiparallel topology. Of note, in a hypothetical antiparallel L15-like fold for L(14,15) or F14L15, residue 14 would be forced in an unfavored *syn* conformation as observed in the formed V-loop structure. Apparently, the energetic penalty for a (*north*,*syn*) conformation of ^LNA^G or ^F^rG strongly depends on the structural context and is better tolerated in the V-loop structure. In fact, while the glycosidic angle in position 14 agrees between V-loop structure and antiparallel fold, the sugar pucker is quite distinct. At the V-loop 5′-end, LNA’s locked C3′-*endo* pucker likely plays a stabilizing role as sugar puckers in the *north* domain are highly conserved among F(14,15), A14F15 and F14L15 (Supplementary Figure S18A) and have also been identified as a recurring motif in monomolecular G4 structures with V-loops (25). In contrast, an S-puckered G14 seems highly beneficial in the third G-tract of the antiparallel L15 and A15 structures (Supplementary Figure S18B), suggesting a larger penalty of a *north*-favoring G14 analog in an antiparallel structure associated with the propensity to form a V-loop structure in case of L(14,15) and F14L15. A strong energetic penalty for a (*north*,*syn*) conformation within a normal G-tract is also reflected in the complete refolding of L15 to the antiparallel topology, avoiding a *syn*-^LNA^G15 as required for the native hybrid-type fold. In contrast, ^F^araG and ^F^rG residues in position 15 were found to be less effective in the structural rearrangement, partially conserving the native topology with a *syn* conformation. However, the latter was stabilized by a sequential F2′· · ·H-C hydrogen bond associated with an S-type sugar pucker, inaccessible for an ^LNA^G residue.

### Base–backbone interactions

A variety of interactions involving C2′-substituents have been proposed to drive the folding of sugar modified DNA G4s but also physiologically more relevant RNA quadruplexes (13,21,26). Thus, especially intraresidual and sequential pseudo-hydrogen bonds with guanine C8H8 are a recurrent structural feature. In contrast, base-backbone interactions such as the here identified contact between ^LNA^G15 O4′ and G14 H8 in the L15 structure seem to be less common. However, the O4′-H8-C8 interaction is highly conserved throughout the structural ensemble of L15 and a similar contact between residues 15 and 16 is found for some states in the F14L15 structure (see above). While observed O4′-H8-C8 angles are rather acute for a potential hydrogen bond, the majority of C–H· · ·O contacts found in RNA structures exhibit angles within this range (53). Interestingly, a similar but intraresidual base-O4′ interaction was observed for pyrimidine H6 protons in Z-DNA and i-motif structures with O4′-H6-C6 angles of around 100° or even lower (54,55). Also, calculations on single C and T residues have confirmed the presence of such contacts for some physiologically relevant conformers, with an indication for both hydrogen bonding and significant energetic contributions of these interactions (56,57).

In spite of a questionable hydrogen bond character of the present O4′· · ·H8–C8 interaction, these contacts may nevertheless locally contribute to the stabilization of a particular motif. In fact, the same O4′-H8 contact as in F14L15 is also highly conserved in the structures of F(14,15) and A14F15 (Supplementary Figure S19B, C). Involvement of the O4′ in an interaction with a neighboring H8 may critically affect G4 binding by preventing crucial O4′ interactions with ligands as often seen for groove binding (58,59). Thus, a hydrogen bond between an end-stacked ligand and O4′ of a 5′-terminal G was suggested to stabilize the corresponding ligand-quadruplex complex (60), pointing to a possible influence of O4′ accessibility also on binding affinities towards G4 structures.

**Figure 8.**
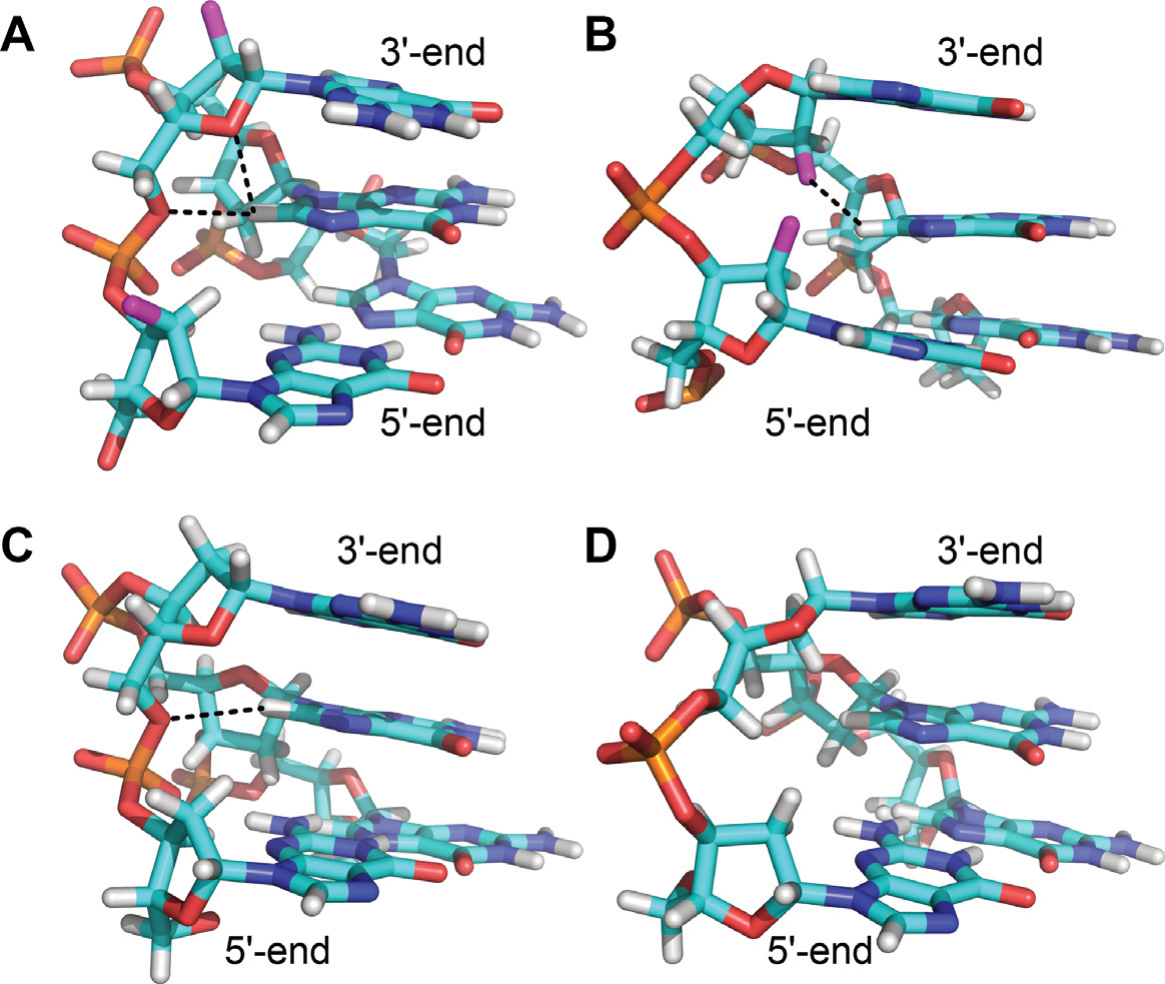
V-loop backbone conformations in different representative G4 structures. V-loop adjacent residues and the following G-tract are shown for F(14,15) (25) (**A**, PDB ID 6RS3), F14A15 (26) (**B**, PDB ID 6TCG), 5ZEV (51) (**C**) and 1U64 (52) (**D**). Residues at the V-loop 5′- and 3′-end are labeled. Short distances between O4′ and/or O5′ of the 3′ V-loop adjacent residue and the following guanine H8 for F(14,15) and 5ZEV (A, C) are in striking contrast to an F2′–H8 interaction in F14A15 (B) and a V-loop backbone that points away from the following G-tract in both F14A15 and 1U64 (B, D).

In F14L15, O4′ and O5′ atoms of ^LNA^G15 apparently compete for the interaction with G16 H8. Of note, in F(14,15) and A14F15, the O4′–H8 contact is conserved throughout the structural ensemble and actually combined with an O5′-H8 interaction in a bifurcated hydrogen bond (Figure 8A). On the other hand, neither O4′ nor O5′ take part in an interaction with G16 H8 in F14A15 where G16 H8 is involved in a pseudo-hydrogen bond with the 2′-F substituent of ^F^araG15 (Figure 8B) (26). The altered G16 H8 interaction(s) reflect the distinct V-loop conformation in F14A15, based on *south*-puckered residues and with a backbone inversion site within the loop. In contrast, only the N-type pucker of residue 15 in combination with the following backbone inversion seems to allow for the unusual O4′ and/or O5′ interaction with G16 H8 in F(14,15), A14F15 and F14L15.

While the O4′ contact is only observed for the three ODN-derived V-loop structures, the O5′ interaction seems to be a recurrent motif in other G4 structures with 0-nt V-shaped loops spanning three tetrad layers. Thus, three unmodified V-loop structures, 5O4D (61), 5ZEV (51) and 2KPR (62) as well as the LNA-modified V4 structure (38) also feature this interaction between O5′ of the V-loop 3′-adjacent residue and H8 of the following G with average distances below or slightly above 3 Å (Figure 8C and Supplementary Figure S20). On the other hand, two bimolecular V-loop structures, 1U64 (52) and 6A7Y (63), as well as the monomolecular G4 6H1K (64) display corresponding distances above 4 Å pointing to an altered V-loop conformation in analogy to F14A15 (Figure 8D). Clearly, the V-loop in spite of being a unique G4 structural motif has many facets and distinct subtypes may be recognized. These are based on subtle differences in interactions and conformations such as the here identified O5′-H8 contact, sugar puckers, backbone orientations, and tetrad polarities and will be discussed in the following section (25,26,65).

### Conformational classification of V-shaped loops

**Figure 9.**
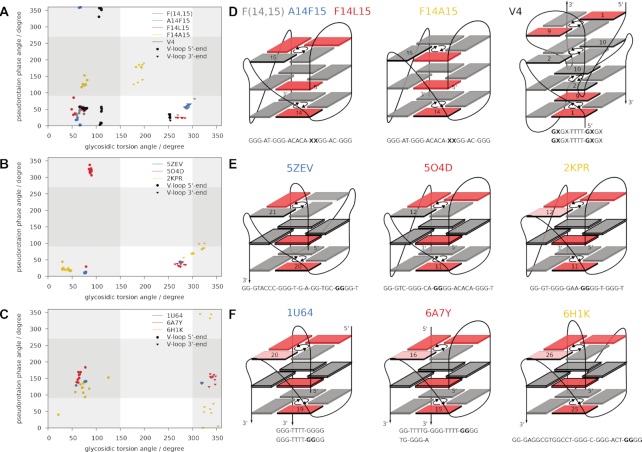
Plots of pseudorotation phase angle vs. glycosidic torsion angle for V-loop flanking residues as well as schematic representations and sequences of corresponding G4 structures. (**A**, **D**) 14,15-modified ODN structures F(14,15) (PDB ID 6RS3) (25), F14L15, A14F15 (PDB ID 6TC8) (26), F14A15 (PDB ID 6TCG) (26), and LNA-modified V4 (PDB ID 2WCN) (38); modified positions in sequences are marked by X. (**B**, **E**) Unmodified V-loop structures 5ZEV (51), 5O4D (61), and 2KPR (62) with exclusively N-puckered V-loop flanking residues. (**C**, **F**) Unmodified V-loop structures 1U64 (52), 6A7Y (63), 6H1K (64) involving *south*-type flanking conformers. (A–C) Vertical and horizontal grey traces indicate areas outside the typical range for *anti* glycosidic torsion angles and *north*-type sugar puckers, respectively. (D–F) *Anti* and *syn* Gs are represented by gray and red rectangles. V-loop residues are labeled, with conformers outside the typical *syn*/*anti* region (low-*syn*) highlighted in light red.

The V-shaped loop, in general linking G-core residues of neighboring antiparallel G-tracts at opposite quadruplex ends, usually involves an isolated *syn* G at the V-loop 5′-end as part of an interrupted G-tract and was first discovered in an interlocked dimeric G-quadruplex (66). As in 14,15-modified ODN, the vast majority of reported V-shaped loops spans three G-quartet layers and consists of zero nucleotides (51,52,61–65), although a few structures with 1-nt or 2-nt V-shaped loops have been described (65,67,68). In an attempt to group reported V-loops into subcategories, Maity *et al.* have proposed a differentiation based on tetrad polarity of the linked G-quartets, with V_R_ and V_S_ loops connecting G-quartets of reverse and same polarity, respectively (65). This classification groups the majority of known V-shaped loops into one class, as the V_R_ type is most common with only few reported V_S_ loops (26,65,67). Based on our findings for mixed ^F^rG/^F^araG modifications in position 14 and 15 of ODN, we have introduced a different classification according to the strand polarity inversion site (26). Thus, F(14,15), A14F15 and F14L15 feature a backbone inversion between V-loop flanking modified residue 15 and G16, whereas the strand polarity inversion occurs within the V-loop in F14A15 with its reversed top tetrad (Figure 9D, left and middle panel). Irrespective of primary sequence and tetrad polarities, this classification differentiates between conventional and alternative V-loops characterized by a backbone inversion site after and within the V-loop, respectively.

*North*-type sugar puckers have previously been identified as a recurring motif for V-loop flanking residues in most monomolecular G4 structures (25). Here, we observe that ^LNA^G with its locked C3′-*endo* sugar pucker has a pronounced stabilizing effect at the V-loop 3′-end and is even tolerated in a very unfavorable *syn* conformation at the V-loop 5′-end. Again, this suggests that sugar conformational preferences may be more important than glycosidic bond angle propensities in the structural context of a V-loop.

To follow common patterns and interrelationships of V-loop conformational parameters, pseudorotation phase angles have been plotted against glycosidic torsion angles in Figure 9. Only 0-nt V-shaped loops spanning three tetrad layers and followed by an uninterrupted G-tract were considered for the analysis. For the modified quadruplexes with incorporated G analogs (Figure 9A), two types of V-loop conformations are immediately apparent. Three conventional 14,15-modified V-loop structures feature conserved *north* sugars at both V-loop anchoring nucleotides, in contrast to the alternative V-loop in F14A15 with S-puckered V-loop adjacent residues (Figure 9A). Extending such plots to unmodified V-loop quadruplexes, two families of V-loop structures can likewise be discriminated: One type exhibits V-loop flanking residues in a *north* conformation (Figure 9B) and the other type features at least one V-loop adjacent residue with a sugar pucker in the *south* domain (Figure 9C).

Apparently, there is a strong correlation between pseudorotation phase and glycosidic torsion angle. In contrast to the (*north*,*north*) type of V-loops with 5′-flanking residues generally adopting an *anti* conformation, V-loop structures with a *south*-puckered residue as shown in Figure 9F exhibit glycosidic torsion angles between 300° and 360° outside the typical *high-anti* range. If we assign the latter torsion angles to *syn*-like (low-*syn* range), all V-loops with sugar conformations in the *south* domain exhibit a polarity inversion within the loop, i.e., between the two anchor nucleotides, whereas (*north*,*north*) V-loops feature a backbone polarity inversion after the V-loop. Thus, our original classification of conventional and alternative V-loops may also be used to discriminate between two distinct sugar conformational clusters of V-shaped loops. Finally, it is also highly beneficial for identifying the presence or absence of intramolecular interactions. In fact, the short O5′-H8 distance between the V-loop 3′-flanking residue and the subsequent G, identified as a characteristic feature of the conventional V-loop in 14,15-modified ODN structures, is mimicked by all other conventional V-loop structures (Supplementary Figure S20). On the other hand, alternative V-loops of Figure 9F exhibit corresponding interatomic distances >4 Å in full analogy to the alternative F14A15 structure.

In conclusion, different characteristic features of conventional and alternative V-loops do not serve as mere formal criteria for the classification, but include important structural details and interactions of possible relevance. In terms of base-backbone interactions, the two distinct V-loop conformational families will differ significantly in their exposure of backbone atoms. As for the above discussed O4′-H8 contacts, the O5′-H8 interaction seen for the conventional V-loop shields the V-loop 3′-adjacent O5′ atom from other intermolecular interactions, contrasting with a fully exposed V-loop backbone in an alternative V-loop. On the other hand, the distinct sugar conformational requirements may allow for a directed manipulation of V-loop type through site-specific incorporation of sugar-modified G analogs. For instance, an ^LNA^G at the V-loop 3′-end will strongly favor a conventional over an alternative V-loop as exemplified by F14L15 and L(14,15).

## CONCLUSION

LNA modifications were effectively used to induce refolding into two distinct non-parallel G4 structures, highlighting LNA’s potential as a versatile tool in quadruplex design. Of note, the enforced C3′-*endo* sugar pucker seems at least as important as LNA’s *anti* preference for local stabilizing effects. In fact, we show for the first time that an energetically most disfavored LNA *syn* conformer may be tolerated at a V-loop 5′-end. On the other hand, a perfectly matching LNA residue at the V-loop 3′-end strongly stabilizes the conventional V-loop structure. The latter is clearly distinguished from an alternative type of V-loop based on *south*-puckered flanking residues and with altered exposure of backbone atoms. LNA substitutions may therefore be used for structural fine-tuning, e.g., by inducing a subtle transition from alternative to conventional type of V-loop with putative impact on the interaction with ligands, proteins, or other receptors.

## DATA AVAILABILITY

The atomic coordinates of the F14L15 and L15 quadruplexes have been deposited in the Protein Data Bank (accession code 6YCV and 6YEP, respectively).

## Supplementary Material

gkaa720_Supplemental_File
