# Supplementary material for: Locked nucleic acid building blocks as versatile tools for advanced G-quadruplex design
Source: Nucleic Acids Res. 2020 Sep 5;48(18):10555–66. doi: 10.1093/nar/gkaa720 (PMC7544228; doi:10.1093/nar/gkaa720)
Supplement: gkaa720_Supplemental_File [file gkaa720_supplemental_file.pdf]

Supplementary Information

Contents

NMR structure calculations **S3**

Resonance Assignment **S3**

Imino resonances of F14L15 . . . . S3
Imino resonances of L(14,15) S4
G-tract assignment for L15 . . . . S4

Supplementary Figures **S5**

Supplementary Tables **S18**

List of Figures

S1 Gel electrophoresis of native ODN and 14,15-modified sequences S5
S2 1D ^{1}H spectra of L14F15 and L14 . . . . . S5
S3 CD spectra of F14L15, L(14,15), and L15 . . . . . S6
S4 Sequential NOE contacts for F14L15 . . . . . S7
S5 H1-H1 and H1-H8 NOE contacts for F14L15 . . . . . S8
S6 H-D exchange experiments for F14L15, L(14,15), and L15 . . . . . S8
S7 H8-H1'/H2' NOE contacts of V-loop flanking residues in F14L15 . . . . . S9
S8 ^{F}rG14 H1'/H3'-H2' NOE crosspeaks for F14L15 in comparison with F(14,15) S9
S9 Sequential NOE contacts for L(14,15) S10
S10 H8-H1'/H2' NOE contacts of V-loop flanking residues in L(14,15) S11
S11 Assignment of imino resonances for L(14,15) S11
S12 Sequential H6/H8-H2'/H2" NOE contacts for L15 . . . . . S12
S13 Sequential H6/H8-H1' NOE contacts for L15 . . . . . S13
S14 H1-H1 and H1-H8 NOE contacts for L15 . . . . . S13
S15 DQF-COSY spectrum of F14L15 . . . . . S14
S16 DQF-COSY spectrum of L15 . . . . . S15
S17 Interatomic distances in F14L15 . . . . . S16
S18 Sugar puckers in ODN derived high-resolution structures S16
S19 Geometric parameters of O4' - H8-C8 interactions in L15, F(14,15), and A14F15 . . . . . S17
S20 O5'-H8 interactions in V-loop structures S17

List of Tables

S1 Overview of 14,15-modified ODN sequences S18
S2 UV melting temperatures of 14,15-modified ODN quadruplexes S19
S3 NMR restraints and statistics for the structure calculations of F14L15 and L15 . . . . . S20
S4 ^{1}H and ^{13}C chemical shifts of F14L15 . . . . . S21
S5 ^{1}H and ^{13}C chemical shifts of L(14,15) S22
S6 ^{1}H and ^{13}C chemical shifts of L15 . . . . . S23

NMR structure calculations

2D NOE crosspeaks were classified as strong (2.9 ± 1.1 Å), medium (4.0 ± 1.5 Å), weak (5.5 ± 1.5 Å), or very weak (6.0 ± 1.5 Å). For exchangeable protons, categories were set to medium (4.0 ± 1.2 Å), weak (5.0 ± 1.2 Å), or very weak (6.0 ± 1.2 Å). In case of strongly overlapped signals, distances were set to 5.0 ± 2.0 Å. Glycosidic torsions were restrained in the range 170–310° or 25–95° for *anti* and *syn* conformers, respectively. The pseudorotation phase angle (PPA) was restricted to 144–180° for experimentally determined (based on DQF-COSY spectra, Figures S15 and S16) *south*-type conformers (all DNA residues except for residues G3 and A9-A13 in F14L15 and G17 and C19 in L15). While the sugar pucker of LNA residues was not restrained, the PPA of ^{F}rG14 in F14L15 was restrained in the range 30–70°. The latter rather narrow restraint was based on the comparison of experimentally determined $^{3}J_{F2'H1'}$ and $^{3}J_{F2'H3'}$ with values predicted using a Karplus-type relationship between vicinal ^{1}H-^{19}F coupling constants and H-C-C-F torsion angles (Figure S8).[1]

For a simulated annealing of the 100 starting structures, a 5 ps equilibration period at 300 K was followed by heating to 1000 K during 10 ps. After 30 ps, the system was cooled to 100 K and finally to 0 K within 45 ps and 10 ps, respectively. Force constants for NMR-derived distance restraints were set to 40 kcal·mol^{-1}·Å^{-2}, for hydrogen bond restraints to 50 kcal·mol^{-1}·Å^{-2}, for glycosidic torsion angle and sugar pucker restraints to 200 kcal·mol^{-1}·rad^{-2}, and for planarity restraints of G-tetrads to 30 kcal·mol^{-1}·Å^{-2}.

For a refinement in water, ten lowest energy structures were neutralized with K^+ ions and two of the cations placed in the center between the eight O6 atoms of two adjacent tetrads. The system was hydrated with TIP3P water molecules in a truncated octahedral box of 10 Å.[2] During initial equilibration, the DNA was fixed with 25 kcal·mol^{-1}·Å^{-2}. After 500 steps of steepest descent and conjugate gradient minimization, the system was heated from 100 to 300 K during 10 ps under constant volume, followed by a decrease in force constants to 5, 4, 3, 2, 1, and 0.5 kcal·mol^{-1}·Å^{-2} and further equilibration. The final simulation of 4 ns duration at 1 atm was performed with restraints only for NMR-derived distances and Hoogsteen hydrogen bonds. The trajectories were subsequently averaged over the last 500 ps and shortly minimized in vacuum for 500 steps.

Resonance Assignment

Imino resonances of F14L15

Central tetrad imino resonances were initially identified by H-D exchange experiments (Figure S6). Two complete sets of inter-tetrad H1-H1 contacts were then easily assigned to ^{F}rG14-G1-^{LNA}G15 and G2-G7-G8 by H1-H8 contacts in the top and central tetrad (Fig. S5). G21 and G22 imino protons are identified by their contacts with G7 and G6 H8, respectively. Their assignment is not only confirmed by the expected H1-H1 contact but also through an inter-tetrad H1-H8 contact between G22 and G7. An anticipated H1-H1 contact between G21 and G6 is not visible as G6 H1, identified by its strong NOE crosspeak to T5 H6, is almost isochronous with G21. Finally, H1-H8 contacts with G21 and G22 identify G16 and G17 H1 resonances, linked by the expected H1-H1 contact and confirmed through an additional inter-tetrad H1-H8 contact between G17 and G21. The remaining imino resonance at 11.94 ppm is assigned to G20 even though it is too broad to give rise to the expected H1-H8 or H1-H1 contact with ^{LNA}G15 and G16. This assignment is confirmed at 30 °C, where the signal sharpens up and the latter contact appears in 2D NOE spectra (not shown).

Imino resonances of L(14,15)

The assignment of the mostly weak and partly very broad imino resonances was accomplished by analysis of only partially observable H1-H1 and H1-H8 NOE correlations with additional support from the comparison to F14L15 imino resonances and H-D exchange experiments (see Fig. S11 and S6). Imino protons of G1 and G7 are identified by their crosspeaks to H8 resonances of G16 and G1, respectively. A complete set of inter-tetrad H1-H1 contacts allowed for the assignment of ^{LNA}G14/^{LNA}G15 and G2/G8 imino resonances from the G1 and G7 H1 assignments, respectively. In agreement with corresponding chemical shifts in F14L15, the two overlapping signals around 11.55 ppm are assigned to G6 and G21 imino protons based on NOE contacts with T5 H6 and G7 H8, respectively. G17 H1 was assigned to the well resolved signal at 11.08 ppm based on the comparison to F14L15, and G16 H1 was subsequently identified through a clear H1-H1 contact with G17. The remaining two signals at 11.95 ppm and 11.40 ppm are too broad to give rise to any correlations in 2D NOE spectra, but were assigned to G20 and G22 again based on the comparison to F14L15.

G-tract assignment for L15

The G14-^{LNA}G15-G16 G-tract is easily recognized by the characteristically downfield shifted H2' resonance of ^{LNA}G15 (Fig. S12). The G6-G7-G8 tract is clearly identified by sequential contacts to the following loop segment (A9-A13) while the first and last G-tracts are unambiguously distinguished by a contact of G3 H8 and H1' to the methyl resonance of T5 (Fig. S12 and S13). G1, G6, G7, G14, G20, and G21 are identified as the six *syn* Gs in the structure. The two *syn-syn-anti* G-tracts are recognized by the reversed $H8_i$-$H1'_{i+1}$ sequential NOE contacts typical for *syn-syn* steps while the two *syn-anti-anti* G-tracts feature a rectangular pattern of sequential H8-H1' correlations for the *syn-anti* steps (Fig. S13).

Supplementary Figures

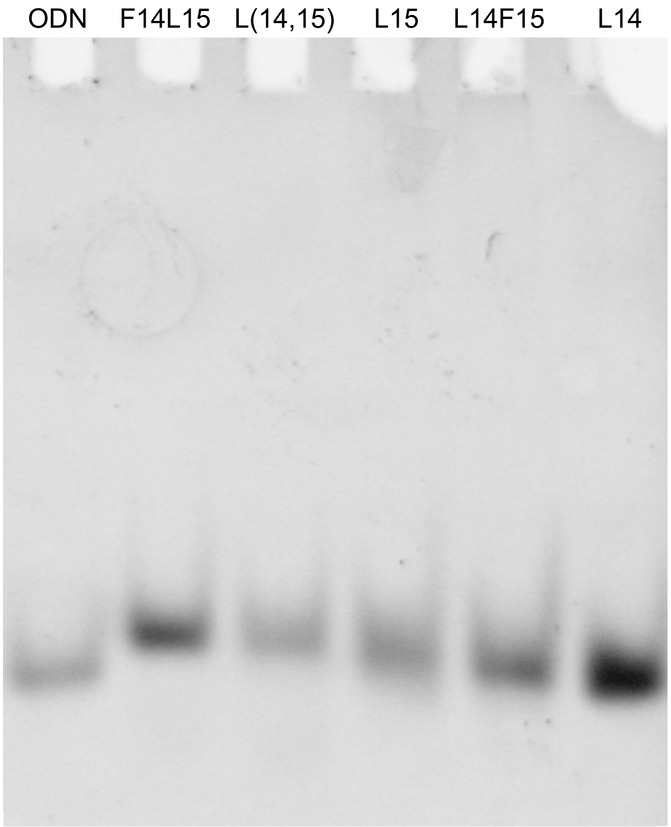

Figure S1: Gel electrophoresis of native ODN and 14,15-modified sequences. The presence of a single band with similar migrating behavior as the native structure confirms the monomeric nature of the modified quadruplexes.

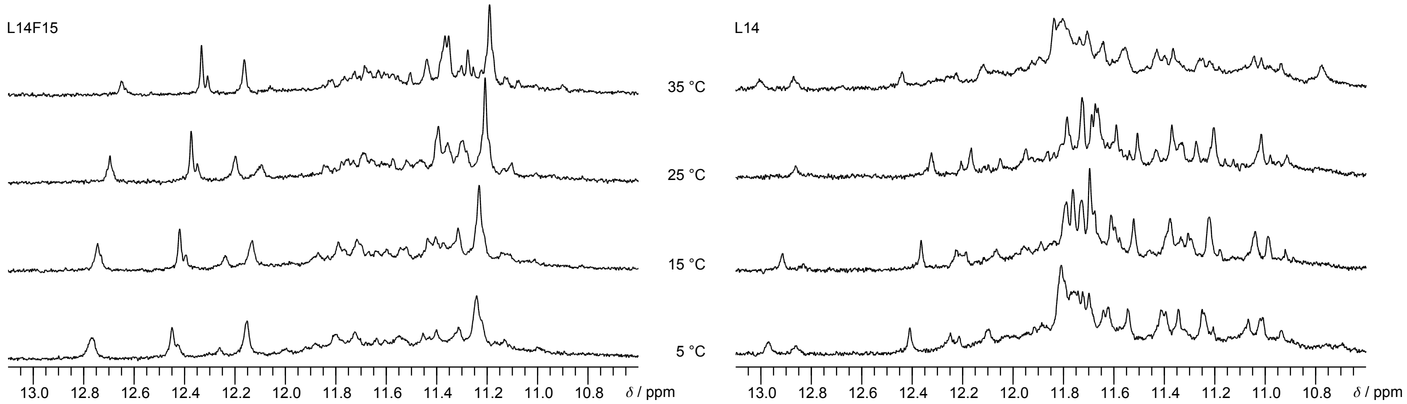

Figure S2: Imino proton spectral region of L14F15 and L14 (0.4 mM) acquired at different temperatures in 10 mM KP_i, pH 7.

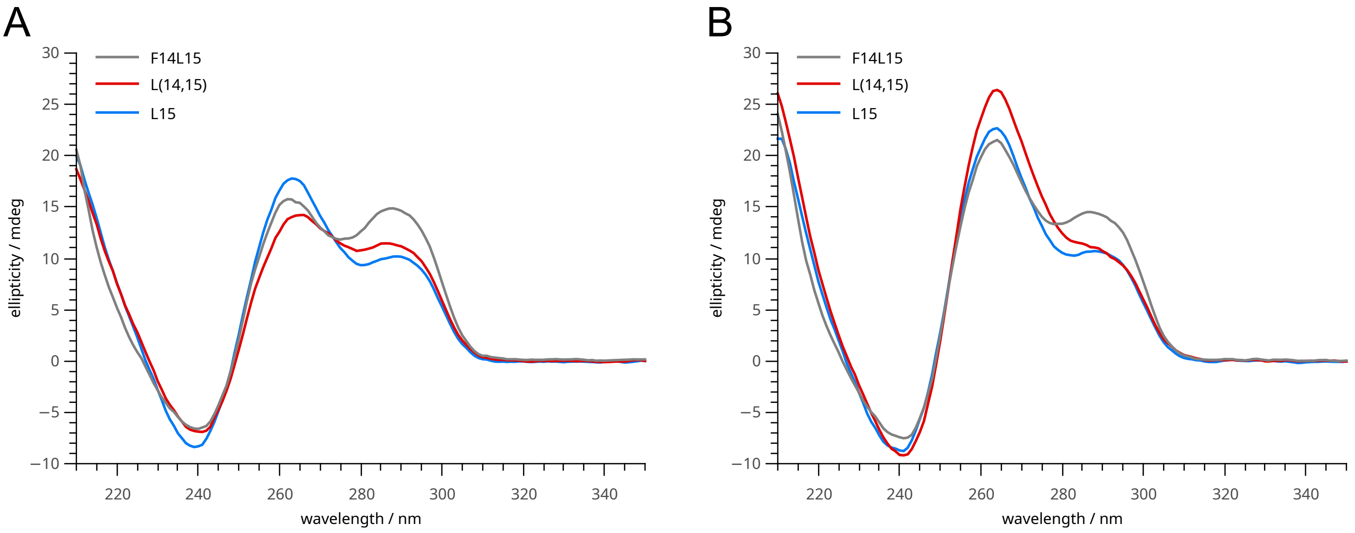

Figure S3: CD spectra of F14L15, L(14,15), and L15 (5 µM) at 25 °C in 10 mM KP_i, pH 7 (A) and 20 mM KP_i, 100 mM KCl, pH 7 (B). All sequences exhibit a negative band around 240 nm along with two positive signals around 263 nm and 290 nm characteristic of a G4 fold featuring both homopolar and heteropolar tetrad stacking. Global structural features seem independent of K^+ concentration as indicated by the presence of the same three bands under both buffer conditions. An increased ellipticity at 263 nm relative to the amplitude at 290 nm in the high-salt buffer as seen for all three structures was previously observed and does not suggest significant structural changes.[3]

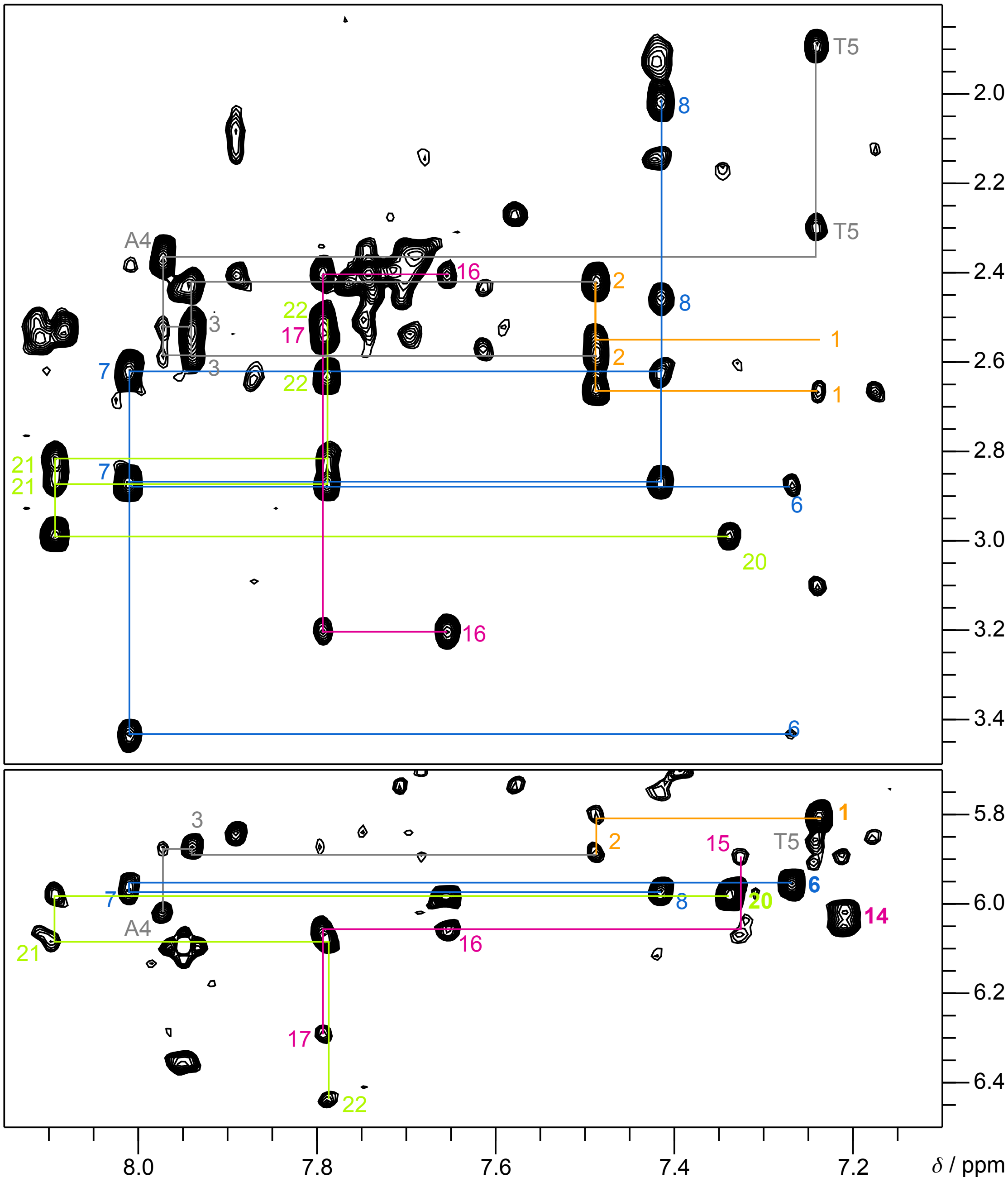

Figure S4: Portions of a 2D NOE spectrum of F14L15 (0.4 mM) acquired at 40 °C in 10 mM KP_i, pH 7. Sequential contacts are traced in different colors for the four G-tracts in the aromatic-H2'/H2" (top) and the aromatic-H1' region (bottom). Contacts extending into loop regions are traced and labeled in grey. Strong H8-H1' crosspeaks for *syn* residues are highlighted in bold.

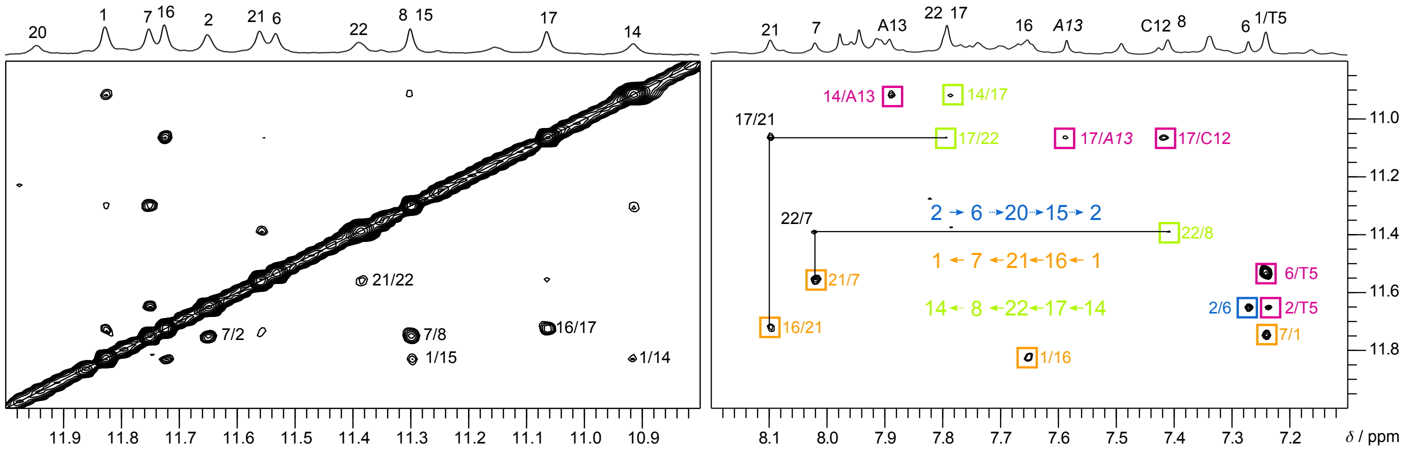

Figure S5: Portions of a 2D NOE spectrum of F14L15 (0.4 mM) acquired at 35 °C in 10 mM KP_i, pH 7, showing $H1(\omega_1)$-$H1(\omega_2)$ (left) and $H1(\omega_1)$-$H8(\omega_2)$ contacts (right) framed in blue, orange, and green for top, central, and bottom tetrad, respectively. Contacts of outer tetrad imino resonances to loop residues are framed in magenta. Adenosine H2 resonances are labeled in italic.

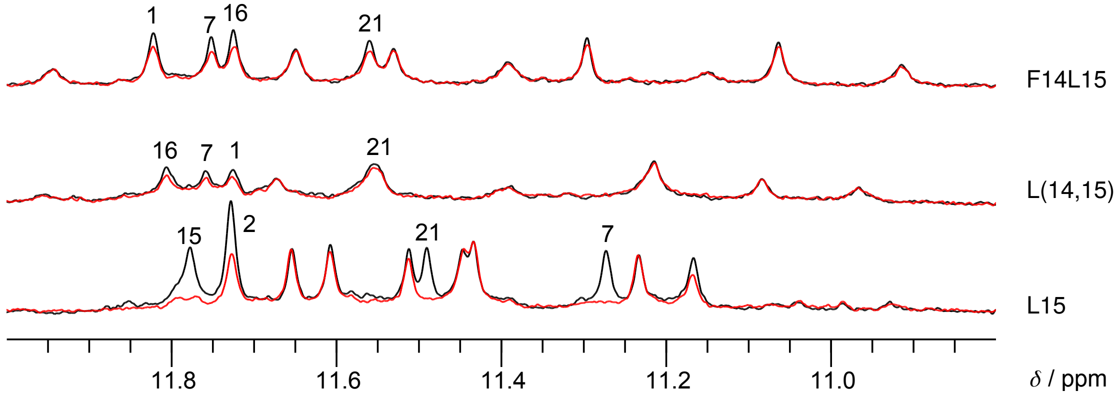

Figure S6: H-D exchange experiments for F14L15 (top), L(14,15) (center), and L15 (bottom). Imino proton spectral region recorded 5 min after redissolving a dried D_2O sample in H_2O (colored) and after full recovery of all signals (in black, after 2 h for F14L15 and L(14,15), after 30 h for L15). Labeled imino protons of the central tetrad are protected from fast solvent exchange and thus identified by their reduced intensities shortly after H_2O addition. Apparently, central tetrad imino protons exchange much faster in the two V-loop topologies when compared to L15.

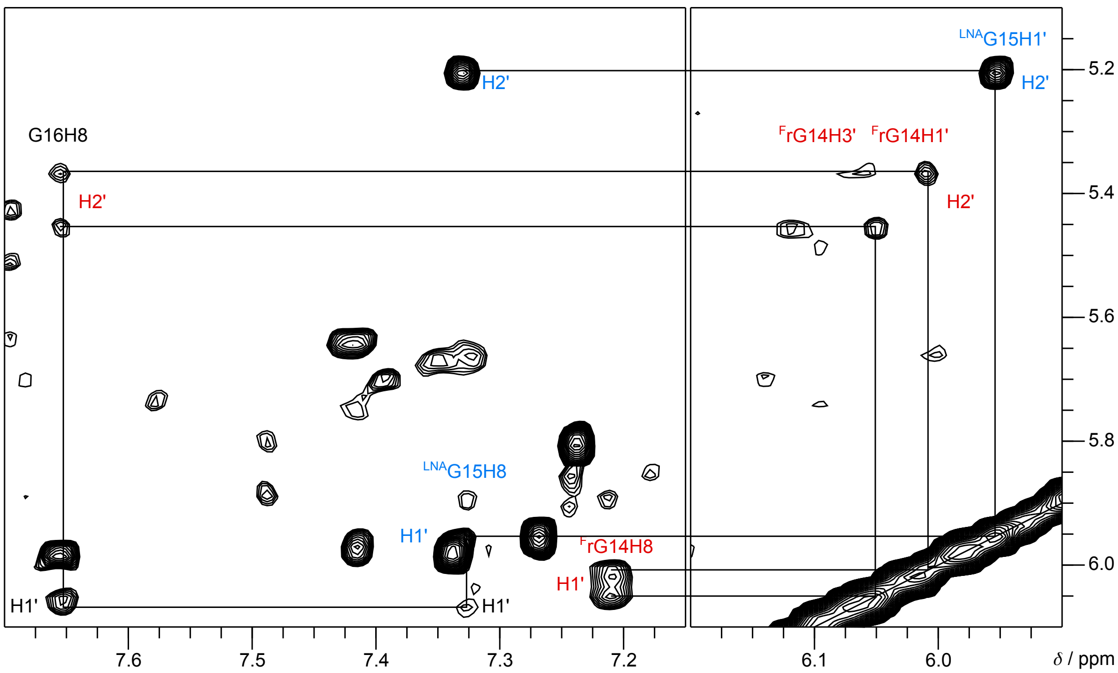

Figure S7: Portions of a 2D NOE spectrum of F14L15 (0.4 mM) acquired at 40 °C in 10 mM KP_i, pH 7, showing intra- and internucleotide contacts of V-loop flanking residues ^{F}rG14 (red) and ^{LNA}G15 (blue) as well as of G16 (black). Contacts between aromatic and sugar protons include the unusual $H8_i$-$H2'_{i-2}$ (G16 H8 - ^{F}rG14 H2') and the reversed sequential $H8_i$-$H1'_{i+1}$ (^{LNA}G15 H8 - G16 H1') contact while a sequential G16 H8 - ^{LNA}G15 H2' crosspeak is clearly missing.

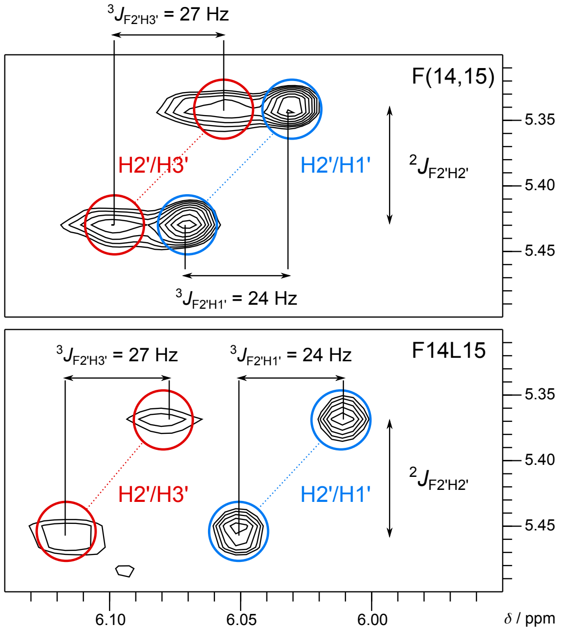

Figure S8: Portions of 2D NOE spectra of F14L15 (bottom) and F(14,15) (top) acquired at 40 °C in 10 mM KP_i, pH 7, showing H2'(ω_1)-H1'/H3'(ω_2) contacts for ^{F}rG14. The E.COSY-type pattern results from vicinal and geminal scalar couplings to ^{19}F2'. An almost identical N-type conformation of ^{F}rG14 in the two stuctures is indicated by perfectly matching $^{3}J_{F2'H1'}$ and $^{3}J_{F2'H3'}$ as well as similarly deshielded H3' resonances.

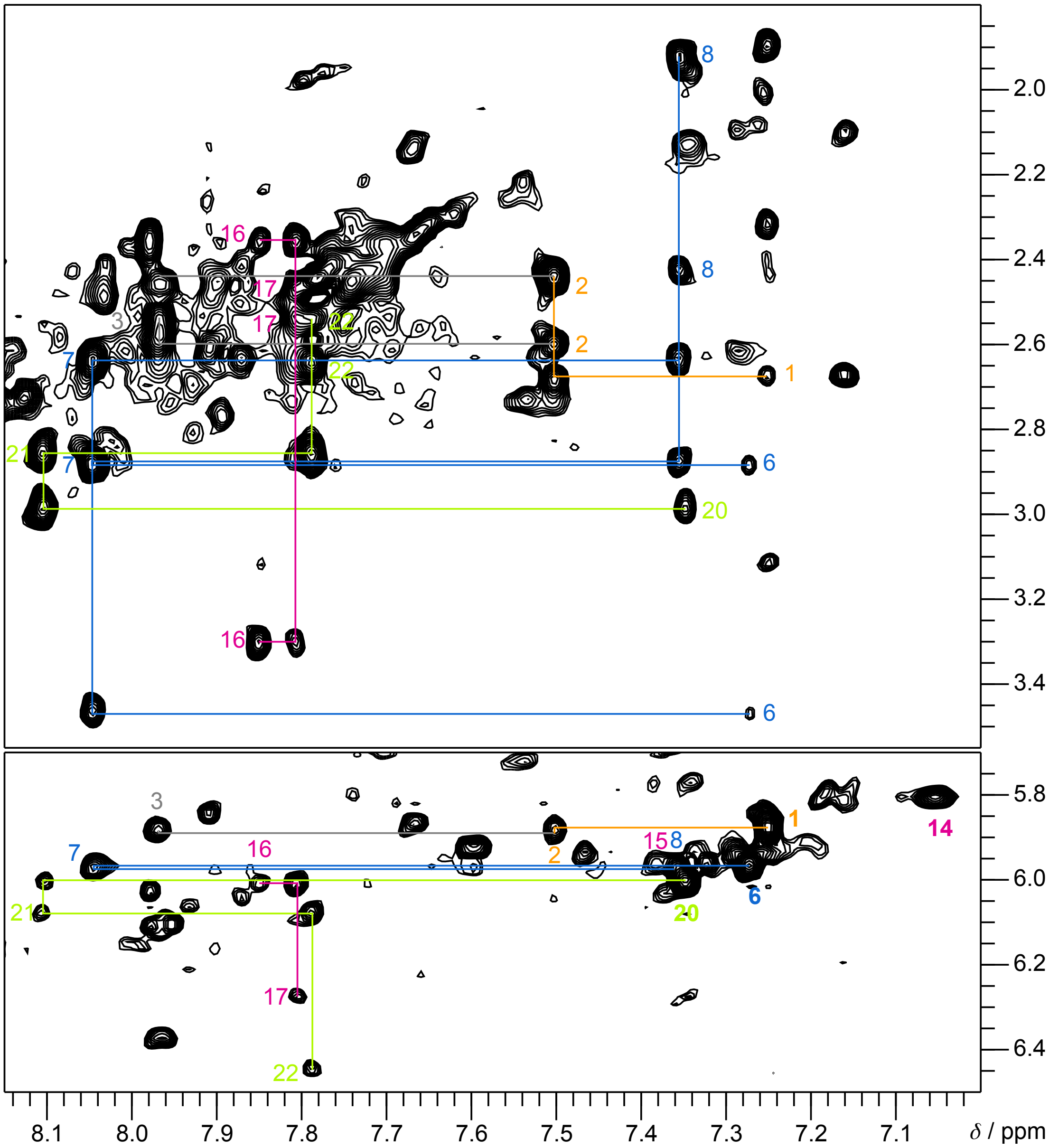

Figure S9: Portions of a 2D NOE spectrum of L(14,15) (0.4 mM) acquired at 30 °C in 10 mM KP_i, pH 7. Sequential contacts are traced in different colors for the four G-tracts in the aromatic-H2'/H2" (top) and the aromatic-H1' region (bottom). Contacts extending into loop regions are traced and labeled in grey. Strong H8-H1' crosspeaks for *syn* residues are highlighted in bold.

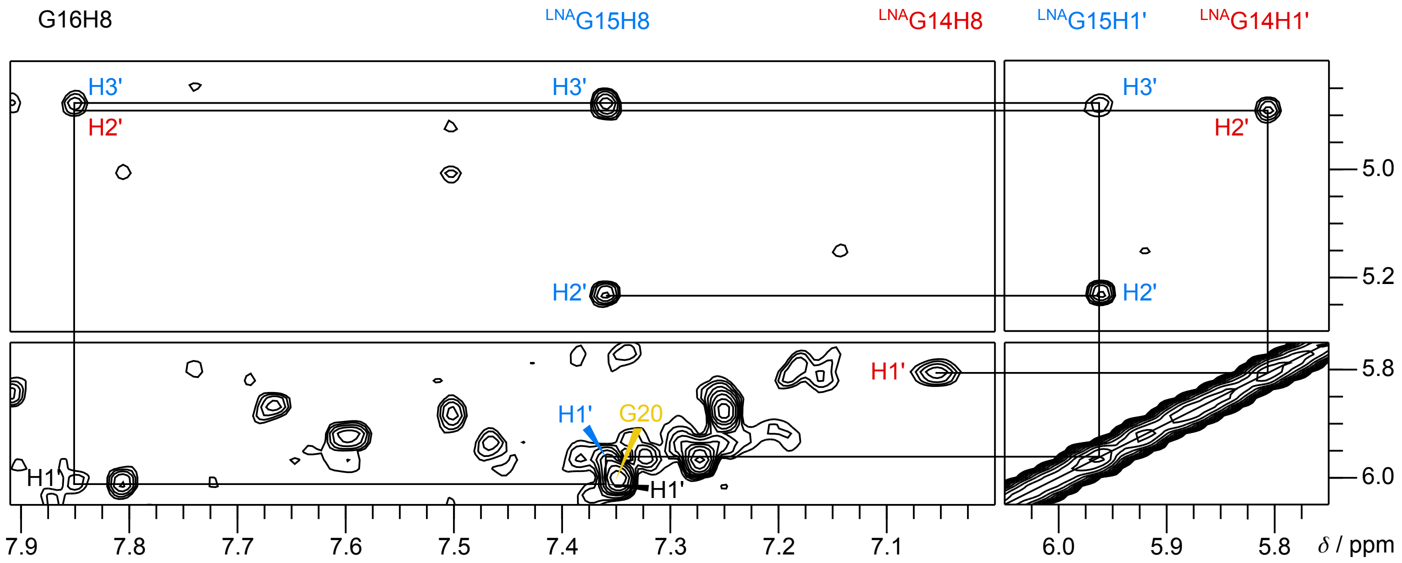

Figure S10: Portions of a 2D NOE spectrum of L(14,15) (0.4 mM) acquired at 30 °C in 10 mM KP$_i$, pH 7, showing intra- and internucleotide contacts of V-loop flanking residues ^{LNA}G14 (red) and ^{LNA}G15 (blue) as well as of G16 (black). Two V-loop characteristic unusual crosspeaks between aromatic and sugar protons, the H8$_i$-H2'$_{i-2}$ (G16 H8 - ^{LNA}G14 H2') and the reversed sequential H8$_i$-H1'$_{i+1}$ (^{LNA}G15 H8 - G16 H1') contact overlap with the sequential G16 H8 - ^{LNA}G15 H3' and the intraresidual G20 H8-H1' NOE crosspeak (labeled in yellow). However, the absence of both an intraresidual H8-H2' contact for ^{LNA}G14 and a sequential G16 H8 - ^{LNA}G15 H2' contact is in agreement with the *syn* conformation of ^{LNA}G14 and a backbone inversion between ^{LNA}G15 and G16, respectively.

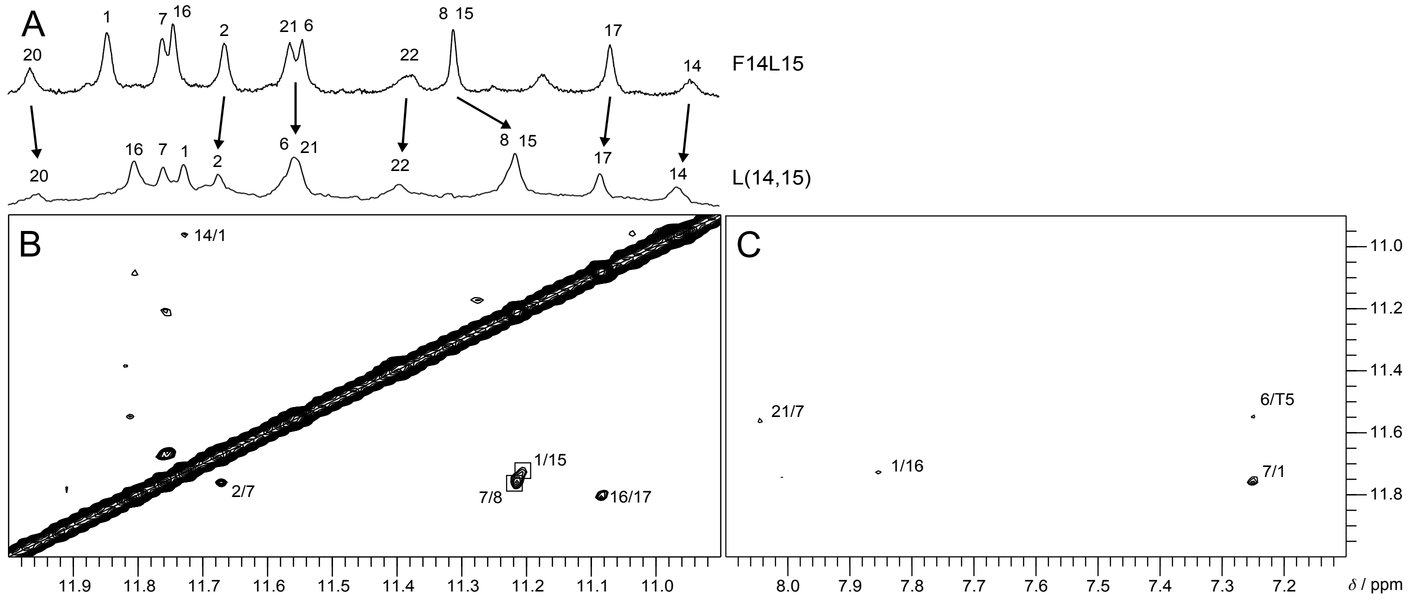

Figure S11: Assignment of imino resonances for L(14,15). A) Imino region of the ^{1}H NMR spectrum of F14L15 (top) and L(14,15) (bottom). Similarities in chemical shift which allowed for the assignment of L(14,15) imino resonances are indicated by arrows. B,C) Portions of a 2D NOE spectrum of L(14,15) showing H1(ω_1)-H1(ω_2) (B) and H1(ω_1)-H6/8(ω_2) contacts (C). All spectra were acquired at 30 °C with a 0.4 mM concentration in 10 mM KP$_i$, pH 7.

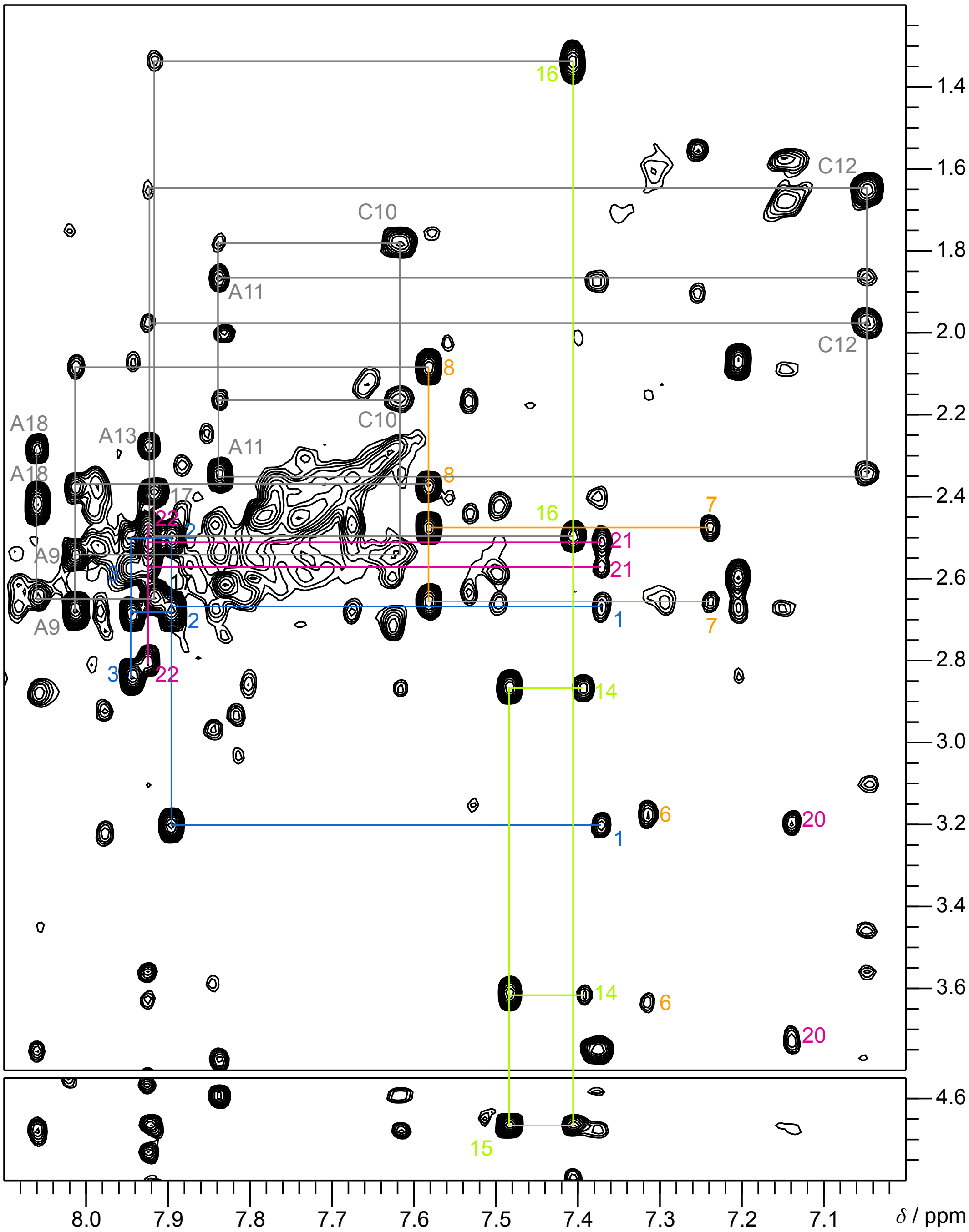

Figure S12: Portion of a 2D NOE spectrum of L15 (0.4 mM) acquired at 25 °C in 10 mM KP_i, pH 7. Sequential H6/H8-H2'/2" contacts are traced in different colors for the four G-tracts. Contacts extending into loop regions are traced and labeled in grey.

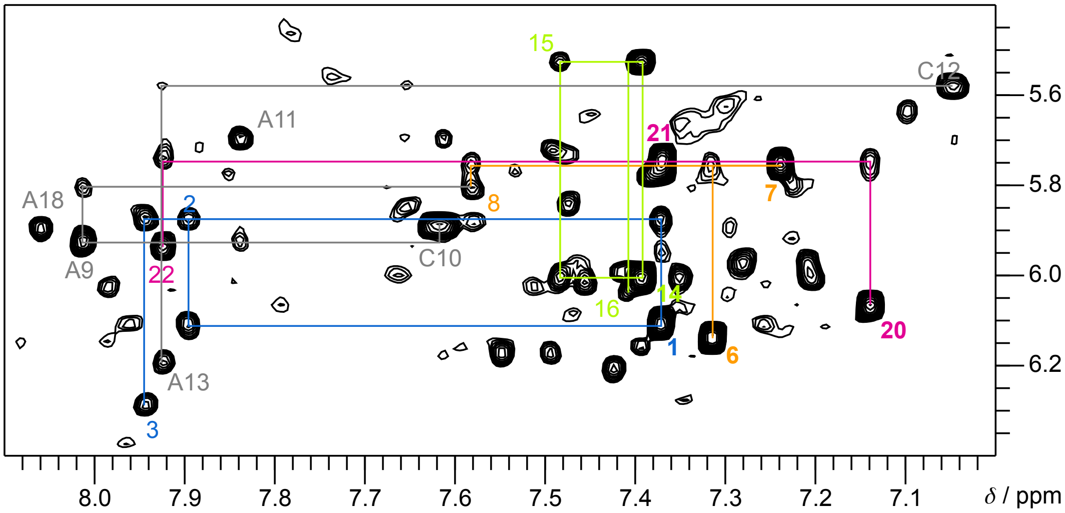

Figure S13: Portion of a 2D NOE spectrum of L15 (0.4 mM) acquired at 25 °C in 10 mM KP_i, pH 7. Sequential H6/H8-H1' contacts are traced in different colors for the four G-tracts. Contacts extending into loop regions are traced and labeled in grey. Strong H8-H1' crosspeaks for *syn* residues are highlighted in bold.

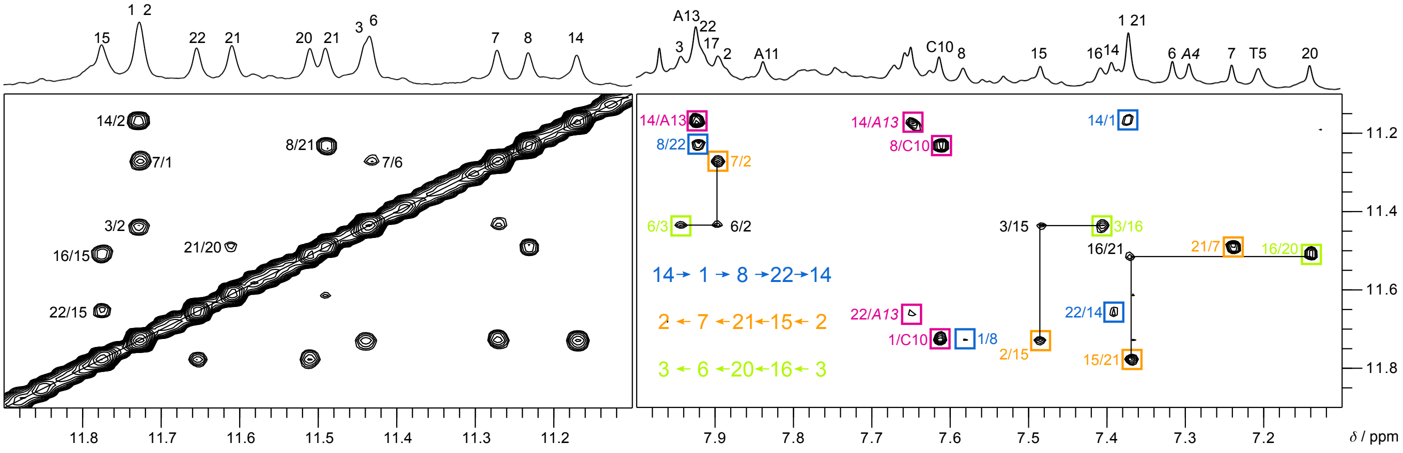

Figure S14: Portions of a 2D NOE spectrum of L15 (0.4 mM) acquired at 25 °C in 10 mM KP_i, pH 7, showing H1(ω_1)-H1(ω_2) (left) and H1(ω_1)-H8(ω_2) contacts (right) framed in blue, orange, and green for top, central, and bottom tetrad, respectively. The resulting hydrogen bond directionality is indicated by arrows. Contacts of outer tetrad imino resonances to loop residues are framed in magenta. Adenosine H2 resonances are labeled in italic.

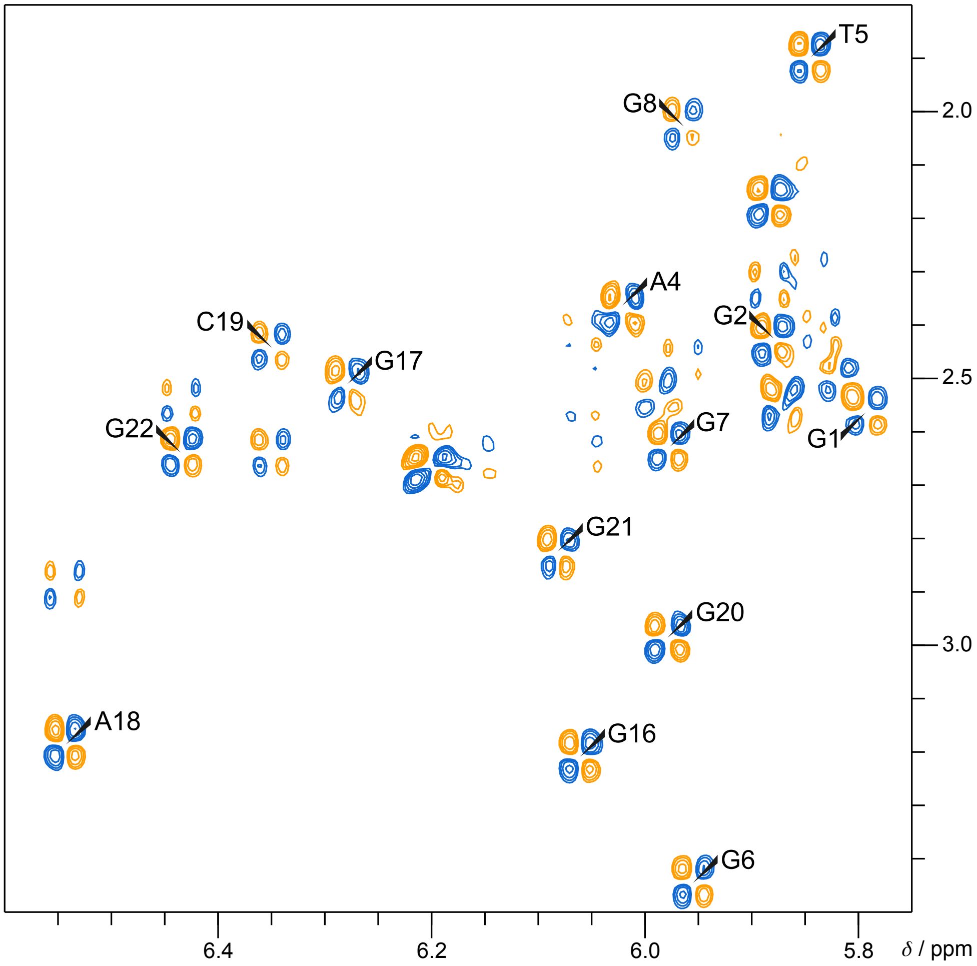

Figure S15: H1'-H2'/H2" region of a DQF-COSY spectrum of F14L15 acquired at 40 °C in 10 mM KP_i, pH 7, 100 % D_2O. Strong H1'-H2' correlations indicative of S-type conformers are labeled.

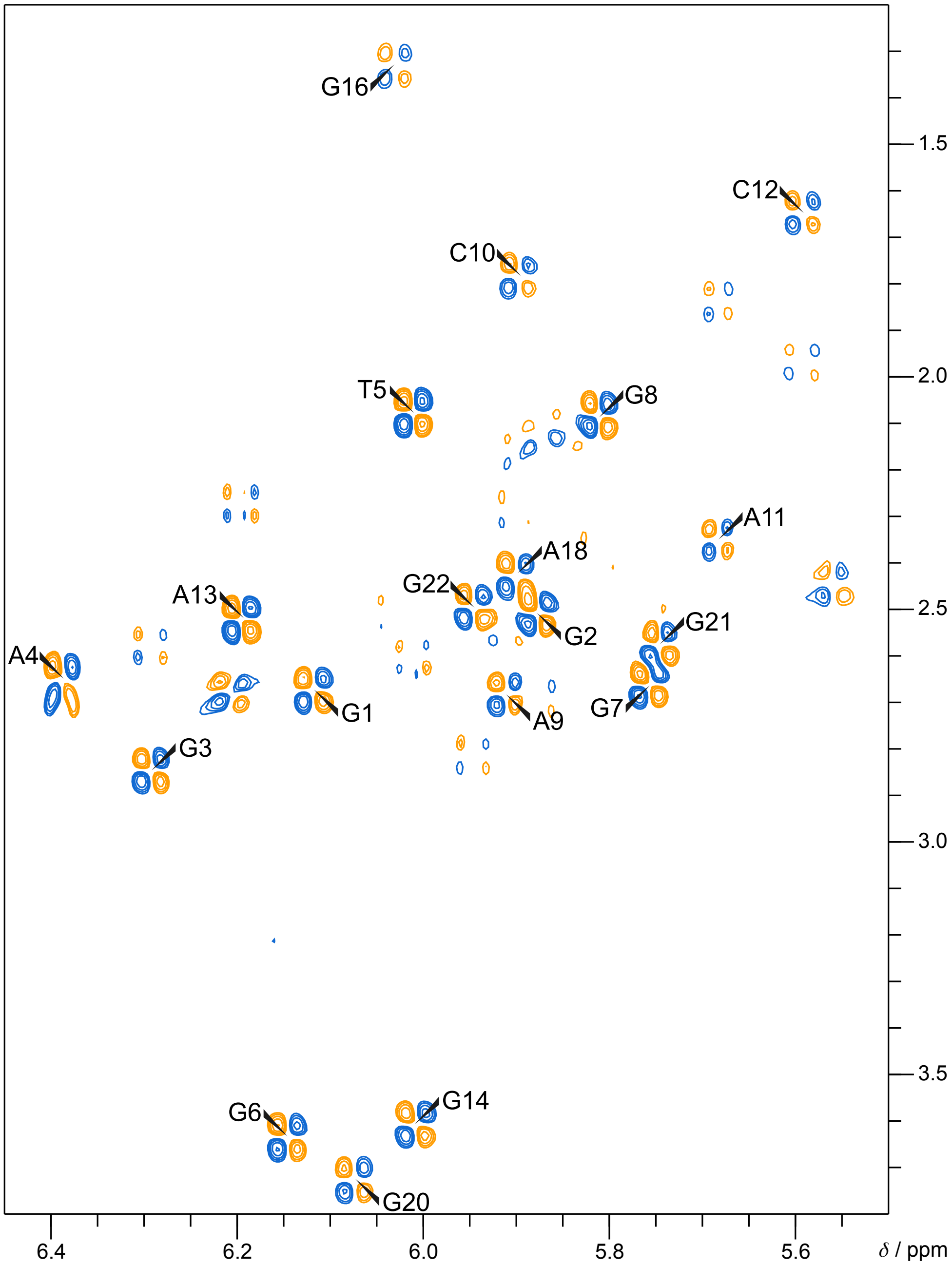

Figure S16: H1'-H2'/H2" region of a DQF-COSY spectrum of L15 acquired at 25 °C in 10 mM KP_i, pH 7, 100 % D_2O. Strong H1'-H2' correlations indicative of S-type conformers are labeled.

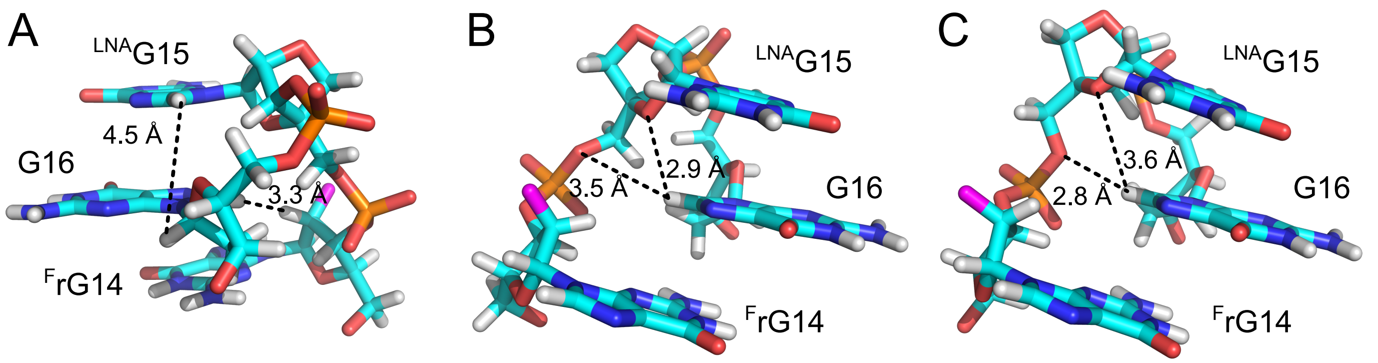

Figure S17: Representative structures of the ^{F}rG14-^{LNA}G15-G16 fragment of F14L15 showing relevant interatomic distances. A) V-loop characteristic short ^{LNA}G15 H8 - G16 H1' and G16 H8 - ^{F}rG14 H2' contacts in agreement with the corresponding NOE crosspeaks. B,C) ^{LNA}G15 O4'/O5' - G16 H8 distances in two different states indicating putative C-H···O hydrogen bonds with participation of O4' (B) or O5' (C).

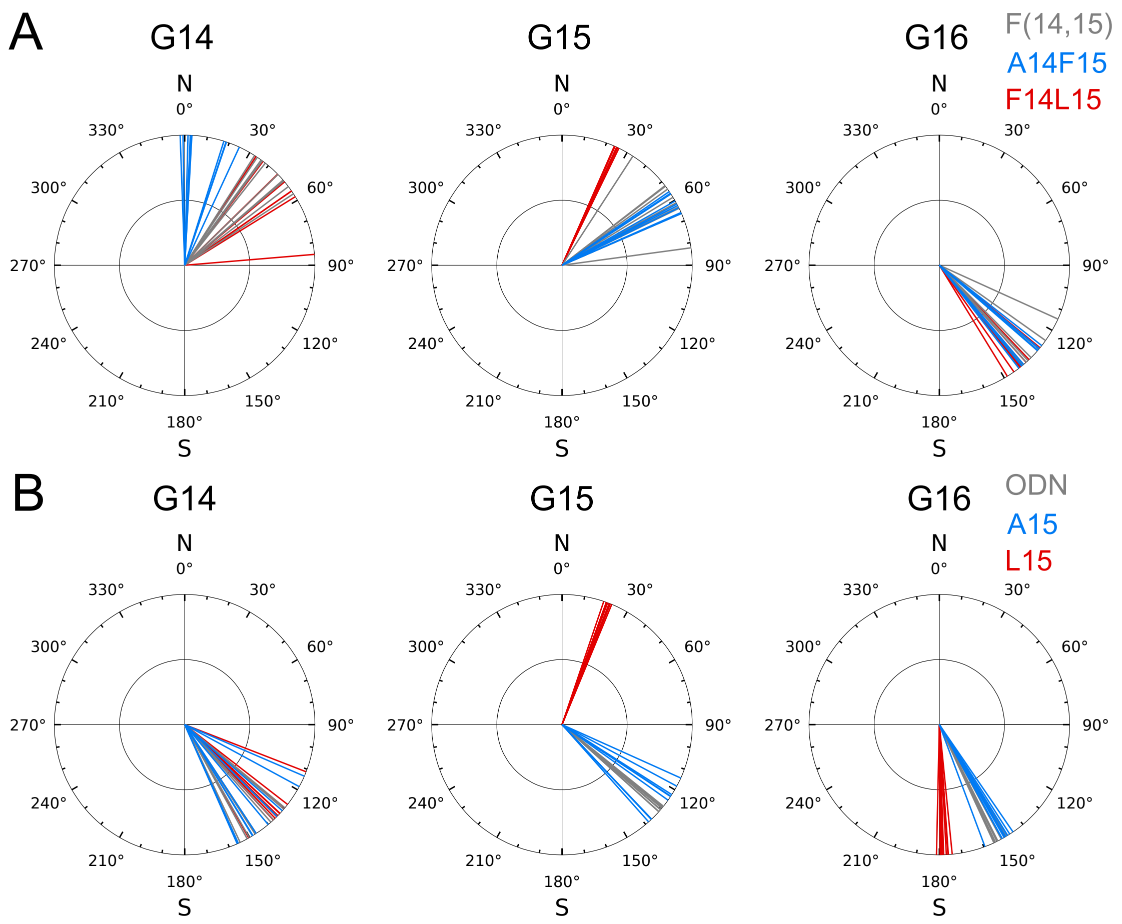

Figure S18: Pseudorotation phase angles of residues 14-16 in (A) F14L15 (red), A14F15[3] (blue, PDB ID 6TC8), and F(14,15)[4] (grey, PDB ID 6RS3) as well as in (B) L15 (red), antiparallel A15[5] (blue, PDB ID 6F4Z), and native ODN[6] (grey, PDB ID 2LOD).

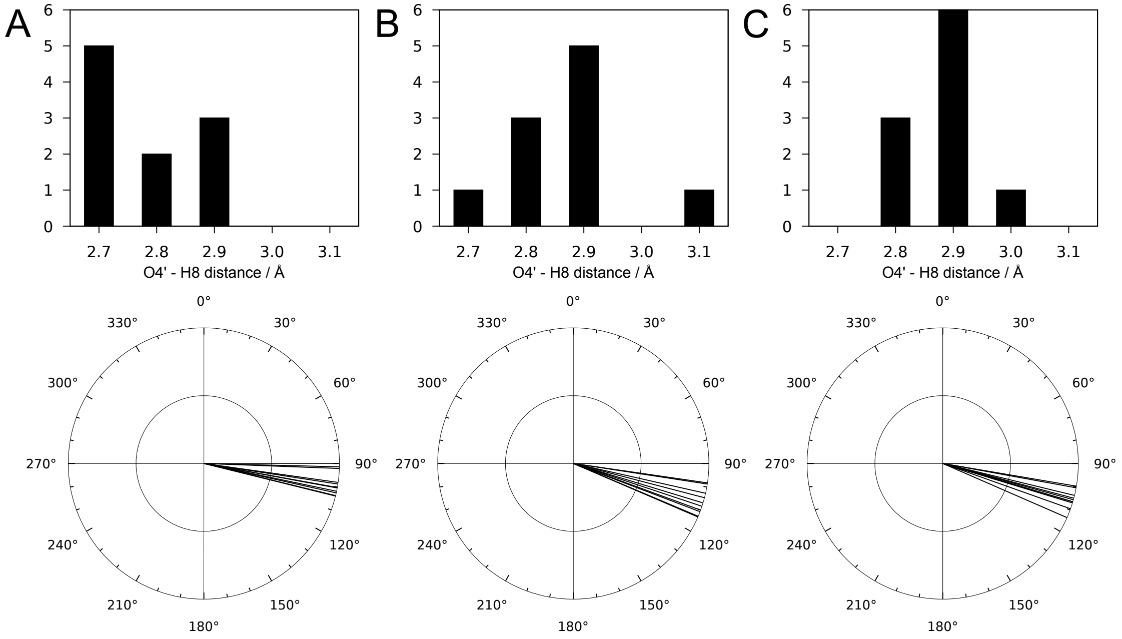

Figure S19: O4' - H8 distances (top) and corresponding O4'-H8-C8 angles (bottom) as extracted from the structural ensembles for the ^{LNA}G15 O4' - G14 H8-C8 interaction in L15 (A) and of the ^{F}rG15 O4' - G16 H8-C8 interaction in F(14,15)[4] (B, PDB ID 6RS3) and A14F15[3] (C, PDB ID 6TC8).

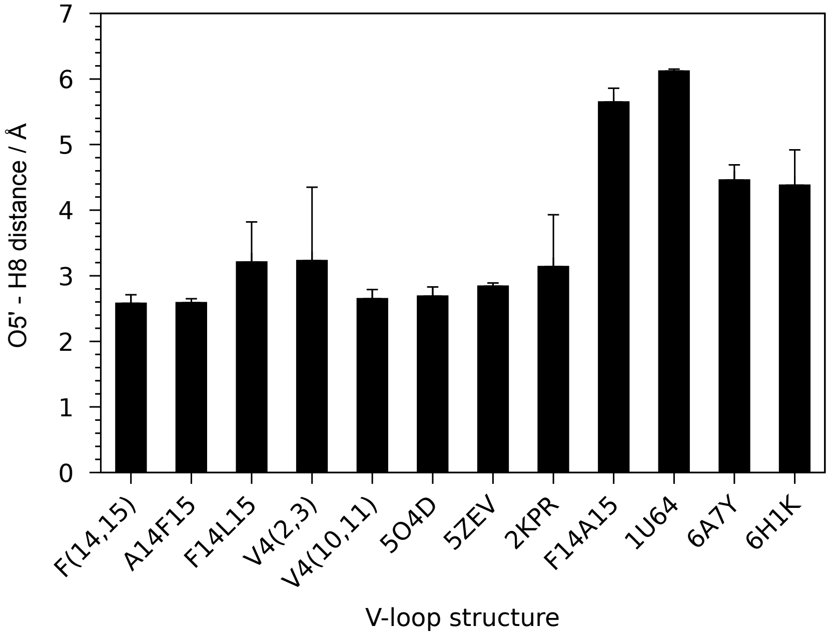

Figure S20: O5'-H8 interactions in V-loop structures. Average distances between O5' of the V-loop 3'-adjacent residue and H8 of the following G-tract residue as extracted from high resolution structures of 14,15-modified ODN,[3,4] the LNA-modified V4 structure[7] (PDB ID 2WCN), and other V-loop featuring G4s (PDB IDs indicated).[8–13]

Supplementary Tables

Table S1: Overview of 14,15-modified ODN sequences, their adopted structures and conformations of introduced G analogs. Unfavored conformations are highlighted in red.[a]

	modifications		adopted structure	conformations of G analogs	
	position 14	position 15	-	position 14	position 15
previous studies					
A15[5]	-	^{F}araG	antiparallel/native	-	(*south*,*anti*/*syn*)
F15[5]	-	^{F}rG	antiparallel/native	-	(*south*,*anti*/*syn*)
F(14,15)[4]	^{F}rG	^{F}rG	V-loop	(*north*,*syn*)	(*north*,*anti*)
r(14,15)[4]	rG	rG	V-loop	(*north*,*syn*)	(*north*,*anti*)
A(14,15)[4]	^{F}araG	^{F}araG	polymorphism	-	-
F14[4]	^{F}rG	-	polymorphism/V-loop	n.d.	-
A14F15[3]	^{F}araG	^{F}rG	V-loop	(*north*,*syn*)	(*north*,*anti*)
F14A15[3]	^{F}rG	^{F}araG	alternative V-loop	(*south*,*syn*)	(*south*,*anti*)
this study					
F14L15	^{F}rG	^{LNA}G	V-loop	(*north*,*syn*)	(*north*,*anti*)
L(14,15)	^{LNA}G	^{LNA}G	V-loop	(*north*,*syn*)	(*north*,*anti*)
L15	^{LNA}G	-	antiparallel	-	(*north*,*anti*)
L14F15	^{LNA}G	^{F}rG	polymorphism	-	-
L14	^{LNA}G	-	polymorphism	-	-

[a] n.d. - not determined

Table S2: UV melting temperatures T_m of 14,15-modified ODN quadruplexes in low-salt and high-salt potassium buffer, pH 7.[a]

	10 mM KP_i		20 mM KP_i, 100 mM KCl	
	T_m / °C	standard deviation	T_m / °C	standard deviation
F(14,15)[4]	45.1	0.5	61.1	0.3
r(14,15)[4]	41.0	0.7	59.2	0.6
A14F15[3]	37.7	0.8	55.8	0.4
F14L15	48.2	0.6	66.4	0.4
L(14,15)	41.6	0.9	59.7	0.2
L15	44.8	0.4	60.5	0.3
L14F15	42.6	0.5	60.4	0.9
L14	41.6	0.2	58.7	0.5

[a]measured in triplicate

Table S3: NMR restraints and structural statistics for the structure calculations of F14L15 and L15.

	**F14L15**	**L15**
**NOE distance restraints**		
intraresidual	71.0	83.0
sequential	59.0	73.0
long-range	35.0	29.0
**other restraints**		
hydrogen bonds	48	48
torsion angles	37	41
**structural statistics**		
pairwise heavy atom RMSD / Å		
G-core	0.54 ± 0.10	0.83 ± 0.16
all residues	3.39 ± 0.76	3.29 ± 0.53
violations / Å		
maximum NOE violation	0.066	0.179
mean NOE violation	0.0012 ± 0.0009	0.0016 ± 0.0007
deviations from idealized geometry		
bonds / Å	0.0107± 0.0001	0.0108 ± 0.0001
angles / degre	2.41 ± 0.02	2.47 ± 0.05

Table S4: ^{1}H and ^{13}C chemical shifts of F14L15 (0.4 mM) at 35 °C in 10 mM KP_i, pH 7.[a]

δ / ppm	H8/H6	C8/C6	H1'	H2'/H2"	H3'	H1	H5/H2/Me
G1	7.24	141.0	5.81	2.55/2.66	4.91	11.83	-
G2	7.49	137.2	5.88	2.42/2.59	4.99	11.65	-
G3	7.94	139.5	5.86	2.51/2.58[b]	4.84	n.d.	-
A4	7.98	141.8	6.02	2.38/2.38	4.74	-	7.79
T5	7.24	139.2	5.85	1.89/2.30	4.63	n.d.	1.53
G6	7.27	142.0	5.96	3.44/2.87	4.86	11.53	-
G7	8.02	138.4	5.98	2.62/2.87	5.04	11.75	-
G8	7.41	137.6	5.96	2.01/2.46	4.90	11.30	-
A9	n.d.	n.d.	n.d.	n.d./n.d.	n.d.	-	n.d.
C10	7.65	143.8	n.d.	n.d./n.d.	n.d.	-	5.98
A11	n.d.	n.d.	n.d.	n.d./n.d.	n.d.	-	n.d.
C12	7.42	143.6	5.77	1.95/2.15[b]	n.d.	-	5.63
A13	7.89	141.4	5.84	2.05/2.42[b]	4.80	-	7.58
^{F}rG14	7.19	140.4	6.03	5.41/-	6.08	10.91	-
^{LNA}G15	7.33	136.1	5.95	5.21/-	4.87	11.30	-
G16	7.65	138.3	6.06	3.21/2.41	4.77	11.72	-
G17	7.80	138.1	6.28	2.51/2.56	5.00	11.06	-
A18	8.55	143.3	6.55	3.18/2.88	4.89	-	8.32
C19	7.95	145.1	6.36	2.45/2.64	n.d.	-	6.09
G20	7.34	141.7	5.99	2.98/2.98	4.82	11.94	-
G21	8.10	138.7	6.08	2.82/2.87	5.09	11.56	-
G22	7.79	138.1	6.44	2.64/2.54	4.74	11.39	-

[a] n.d. - not determined

[b] no stereospecific assignment

Table S5: ^{1}H (at 30 °C) and ^{13}C chemical shifts (at 35 °C) of L(14,15) (0.4 mM) in 10 mM KP_i, pH 7.[a]

δ / ppm	H8/H6	C8/C6	H1'	H2'/H2"[b]	H1	H5/H2/Me
G1	7.25	140.8	5.88	2.68/n.d.	11.73	-
G2	7.50	137.2	5.89	2.44/2.59	11.67	-
G3	7.97	139.5	5.88	2.53/2.59	n.d.	-
A4	7.98	141.8	6.03	2.34/2.38	-	7.77
T5	7.25	139.1	5.85	1.89/2.31	n.d.	1.56
G6	7.27	142.0	5.97	2.88/3.47	11.55	-
G7	8.05	138.3	5.98	2.64/2.88	11.76	-
G8	7.35	137.4	5.97	1.92/2.43	11.21	-
A9	n.d.	n.d.	n.d.	n.d./n.d.	-	n.d.
C10	7.60	143.7	5.97	2.06/n.d.	-	5.92
A11	n.d.	n.d.	n.d.	n.d./n.d.	-	n.d.
C12	7.34	143.6	n.d.	n.d./n.d.	-	5.56
A13	7.91	141.6	5.84	2.61/n.d.	-	n.d.
^{LNA}G14	7.06	141.3	5.81	4.89/-	10.96	-
^{LNA}G15	7.36	136.1	5.96	5.23/-	11.21	-
G16	7.85	139.3	6.01	2.36/3.30	11.80	-
G17	7.81	138.2	6.27	2.47/2.54	11.08	-
A18	8.56	143.2	6.56	2.90/3.17	-	n.d.
C19	7.97	145.0	6.37	2.46/2.66	-	6.11
G20	7.35	141.6	6.00	2.99/2.99	11.95	-
G21	8.10	138.6	6.08	2.86/2.86	11.56	-
G22	7.79	138.1	6.44	2.53/2.64	11.40	-

[a] n.d. - not determined

[b] no stereospecific assignment

Table S6: ^{1}H and ^{13}C chemical shifts of L15 (0.4 mM) at 25 °C in 10 mM KP$_i$, pH 7.[a]

δ / ppm	H8/H6	C8/C6	H1'	H2'/H2"[b]	H3'	H1	H5/H2/Me
G1	7.37	141.1	6.11	2.67/3.20	5.02	11.72	-
G2	7.89	138.4	5.87	2.50/2.68	5.12	11.73	-
G3	7.94	137.0	6.29	2.84/2.57	5.01	11.44	-
A4	8.30	142.2	6.39	2.64/2.67[b]	5.03	-	7.29
T5	7.20	137.9	6.00	2.07/2.60	4.87	n.d.	1.11
G6	7.31	141.6	6.14	3.63/3.18	4.86	11.43	-
G7	7.24	140.4	5.75	2.65/2.48	5.00	11.27	-
G8	7.58	137.7	5.80	2.08/2.37	4.93	11.23	-
A9	8.01	141.7	5.93	2.67/2.55	4.94	-	n.d.
C10	7.62	143.7	5.88	1.78/2.16	4.59	-	5.89
A11	7.84	141.4	5.70	2.34/1.86	4.35	-	n.d.
C12	7.05	143.5	5.58	1.65/1.98	4.39	-	5.11
A13	7.92	141.6	6.19	2.52/2.28	4.67	-	7.65
G14	7.39	141.3	6.00	3.62/2.87	4.94	11.17	-
G15	7.48	137.2	5.53	4.66/-	4.34	11.77	-
G16	7.41	136.8	6.03	1.34/2.50	4.79	11.51	-
G17	7.91	138.5	6.03	2.64/2.39	n.d.	n.d.	-
A18	8.06	142.3	5.90	2.42/2.29	n.d.	-	n.d.
C19	7.38	143.1	n.d.	1.87/2.40[b]	n.d.	-	5.78
G20	7.14	141.3	6.07	3.20/3.73	4.82	11.61	-
G21	7.37	140.9	5.74	2.51/2.57[b]	5.04	11.49	-
G22	7.92	138.7	5.94	2.47/2.80	4.73	11.65	-

[a] n.d. - not determined

[b] no stereospecific assignment

References

[1] C. Thibaudeau, J. Plavec and J. Chattopadhyaya, *J. Org. Chem.*, 1998, **63**, 4967–4984.

[2] W. L. Jorgensen, J. Chandrasekhar, J. D. Madura, R. W. Impey and M. L. Klein, *J. Chem. Phys.*, 1983, **79**, 926–935.

[3] L. Haase and K. Weisz, *Chem. Commun.*, 2020, **56**, 4539–4542.

[4] L. Haase, J. Dickerhoff and K. Weisz, *Chem. Eur. J.*, 2020, **26**, 524–533.

[5] J. Dickerhoff and K. Weisz, *ChemBioChem*, 2018, **19**, 927–930.

[6] M. Marušič, P. Šket, L. Bauer, V. Viglasky and J. Plavec, *Nucleic Acids Res.*, 2012, **40**, 6946–6956.

[7] J. T. Nielsen, K. Arar and M. Petersen, *Angew. Chemie - Int. Ed.*, 2009, **48**, 3099–3103.

[8] M. Marušič and J. Plavec, *Molecules*, 2019, **24**, 1294.

[9] Y. Liu, W. Lan, C. Wang and C. Cao, *J. Biol. Chem.*, 2018, **293**, 8947–8955.

[10] V. Kuryavyi and D. J. Patel, *Structure*, 2010, **18**, 73–82.

[11] C. Wan, W. Fu, H. Jing and N. Zhang, *Nucleic Acids Res.*, 2019, **47**, 1544–1556.

[12] M. Črnugelj, P. Šket and J. Plavec, *J. Am. Chem. Soc.*, 2003, **125**, 7866–7871.

[13] E. Butovskaya, B. Heddi, B. Bakalar, S. N. Richter and A. T. Phan, *J. Am. Chem. Soc.*, 2018, **140**, 13654–13662.
